# Antioxidant metabolites from the endophytic fungus *Aspergillus spelaeus*

**DOI:** 10.1007/s13659-026-00637-9

**Published:** 2026-11-02

**Authors:** Jia-Tong Zhou, Min Wang, Qian Wu, Min Liang, Wei-Feng Xu, Jun Li, Li-Jun Zhang, Rui-Yun Yang

**Affiliations:** Key Laboratory for Chemistry and Molecular Engineering of Medicinal Resources (Ministry of Education of China), Guangxi Key Laboratory of Chemistry and Molecular Engineering of Medicinal Resources, University Engineering Research Center for Chemistry of Characteristic Medicinal Resources (Guangxi), School of Chemistry and Pharmaceutical Sciences, Guangxi Normal University, Guilin, 541004 People’s Republic of China

**Keywords:** *Aspergillus spelaeus*, drimane-type sesquiterpenoids, Amide, antioxidant activity, neuroprotective effects

## Abstract

Five undescribed compounds, including two drimane-type sesquiterpenoids, asperdrimanes A and B (**1** and **2**), and three amide compounds, asperspamides A–C (**3**–**5**), together with four known metabolites **6**–**9**, were isolated from the fungus *Aspergillus spelaeus* GDGJ-286. By extensively analyzing NMR, LC–MS/MS, Marfey's method, and X-ray crystallography, the structures were characterized successfully. Asperdrimanes A and B (**1** and **2**) are drimane-type sesquiterpenoids characterized by a spiro-*β*-lactone moiety. Asperspamide B (**4**) exhibited exceptional antioxidant capacity in ABTS, FRAP, and DPPH tests, along with significant neuroprotective effects in a hydrogen peroxide-induced PC12 cell oxidative injury model, demonstrating strong anti-apoptotic and anti-necrotic properties. These findings highlight asperspamide B’s potential as a lead compound for developing antioxidant agents.

**Graphical abstract:**

**Figure:**
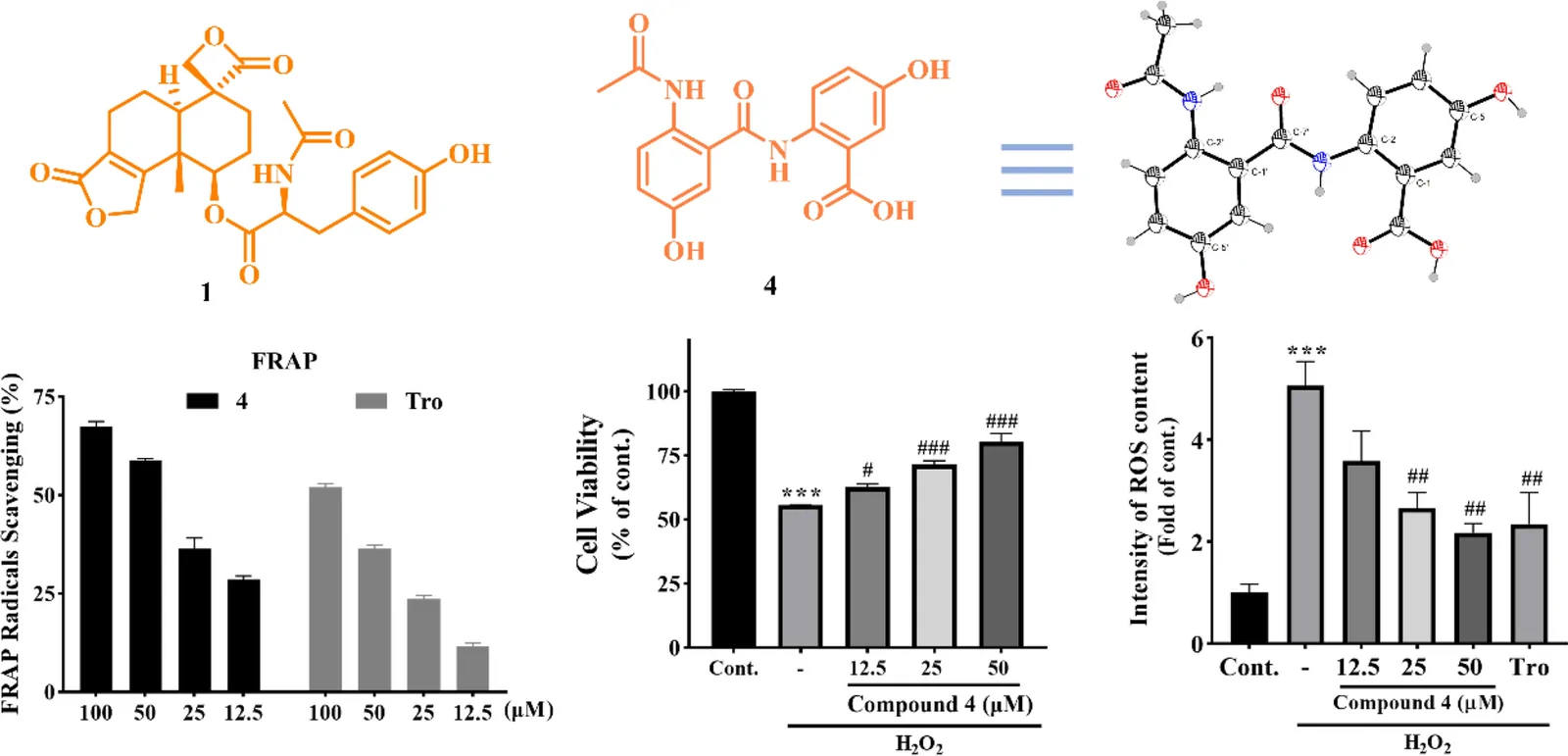

**Supplementary Information:**

The online version contains supplementary material available at https://doi.org/10.1007/s13659-026-00637-9.

## Introduction

Oxidative damage occurs when the balance between oxidant production and the body's antioxidant defense is disrupted [1, 2]. This imbalance can be triggered by both endogenous metabolic processes, including inefficient oxidative phosphorylation within mitochondria, which may generate excessive reactive oxygen species (ROS) leading to mitochondrial dysfunction, and exogenous factors such as environmental toxins. ROS, at low levels, play vital roles in immune function, cellular signaling, and vasomodulation. Nevertheless, once their concentration surpasses the body's antioxidant capacity, they damage lipids, proteins, and nucleic acids, leading to metabolic disruption [3, 4]. Because of its high lipid content and oxygen consumption, the central nervous system is susceptible to oxidative damage [5]. Oxidative stress contributes to neurodegenerative disorders (e.g., Alzheimer's disease and Parkinson's disease) by promoting biochemical alterations in neuronal components and by contributing to mitochondrial dysfunction and neuroinflammation [6]. These oxidative insults ultimately lead to cellular macromolecular damage, necrosis, and apoptotic cell death, highlighting the importance of oxidative stress in neurodegenerative disorders, as well as the potential therapeutic benefits of antioxidants.

Fungi, a diverse and ecologically crucial group, are renowned for producing numerous specialized metabolites with varied bioactivities [7–9]. Among them, the genus *Aspergillus,* which is widely distributed across terrestrial and marine ecosystems [10, 11], has emerged as a highly promising reservoir for discovering novel bioactive specialized metabolites [12–14], especially in antioxidant activity. For instance, asplactones A, B, and D, unique diphenyl ether hybrids isolated from *Aspergillus* sp. F1-8A, exhibited potent DPPH radical scavenging activity, outperforming the synthetic standard BHT [15]. Furthermore, (+)-4,7-dihydroxymellein, isolated from *Aspergillus ochraceus*, demonstrated superior antioxidant and neuroprotective efficacy by dose-dependently mitigating H_2_O_2_-induced oxidative injury in SH-SY5Y cells through upregulation of glutathione and scavenging of ROS [16].

Our research group is engaged in discovering novel bioactive natural products from fungi [17–20]. In our recent investigations, the fungus *Aspergillus spelaeus* GDGJ-286 has emerged as a prolific producer of structurally diverse specialized metabolites. Specifically, this strain yielded two cytotoxic sulfur-containing heterodimers, sucurchalasins A and B, comprising a cytochalasan and a macrolide moiety, alongside a series of cytochalasan monomers and macrolide derivatives [21]. Based on these findings, we proceeded to further investigate the metabolic potential of *A. spelaeus* GDGJ-286. Subsequent chemical investigation resulted in two undescribed drimane-type sesquiterpenoids, asperdrimanes A and B (**1** and **2**), in company with three new amides, asperspamides A–C (**3**–**5**). Additionally, four known metabolites, methyl-3,4,5-trimethoxy-2-(2-(nicotinamido)benzamido)benzoate (**6**) [22], as well as three peptides, WIN 66306 (**7**) [23], WIN 68577 (**8**) [23], and nectriatide (**9**) [24], were also isolated. Asperdrimane A (**1**) represents the first example of a hybrid compound comprising a drimane-type sesquiterpene core and an *N*-acetyl tyrosine moiety to be isolated from nature. Here, the isolation, structural elucidation, and antioxidant activities of these compounds are presented.

## Results and discussion

### Structure elucidation

**Fig. 1:**
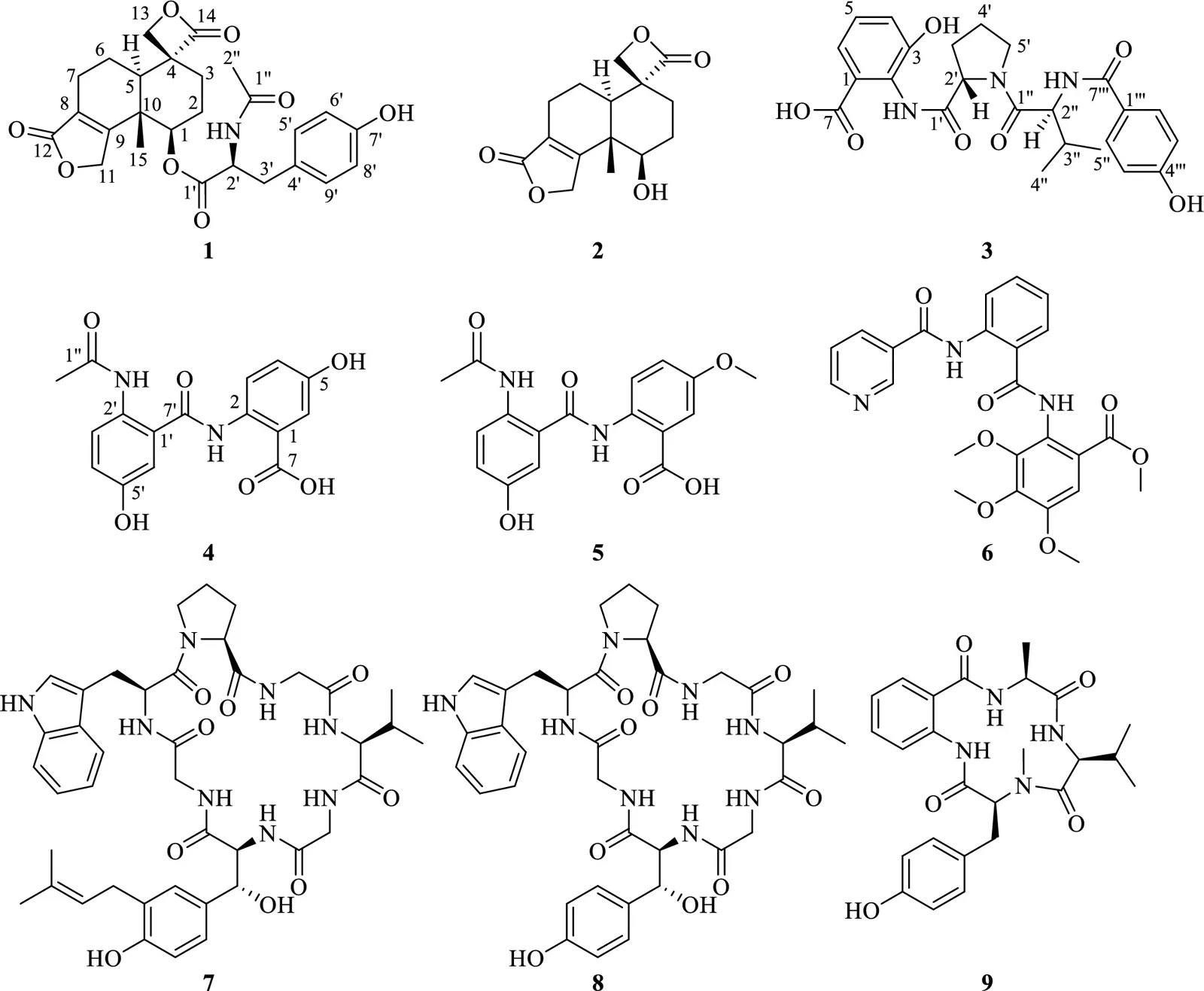
Structures of compounds** 1**–**9**

Asperdrimane A (**1**) (colorless crystals) was assigned a molecular formula of C_26_H_29_NO_8_ (Ω = 13) from the HR-ESIMS ion at *m/z* 484.1962 [M + H]^+^ (calcd. for C_26_H_30_NO_8_^+^, 484.1966). The ^1^H NMR spectrum (Table 1) of **1** indicated the presence of two methyls at *δ*_H_ 1.83 (s, 3H, H-2′′) and 0.90 (s, 3H, H-15); seven sets of methylene protons at *δ*_H_ 4.92 (dt, *J* = 18.0, 2.2 Hz, 1H, H-11a), 4.71 (dd, *J* = 18.0, 2.2 Hz, 1H, H-11b), 4.51 (d, *J* = 5.6 Hz, 1H, H-13a), 4.30 (d, *J* = 5.6 Hz, 1H, H-13b), 2.82 (d, *J* = 7.6 Hz, 2H, H-3′), 2.25 (d, *J* = 16.8 Hz, 1H, H-7a), 2.09 (overlapped, 2H, H-3a, H-7b), 1.98 (overlapped, 1H, H-6a), 1.81 (d, *J* = 4.0 Hz, 1H, H-2a), 1.77 (dd, *J* = 13.8, 4.0 Hz, 1H, H-3b), 1.51 (dd, *J* = 10.6, 7.4 Hz, 1H, H-6b), and 1.35 (m, 1H, H-2b); three methines at *δ*_H_ 4.63 (dd, *J* = 10.8, 4.0 Hz, 1H, H-1), 4.35 (dd, *J* = 14.4, 7.6 Hz, 1H, H-2′), and 1.98 (overlapped, 1H, H-5); four aromatic protons at *δ*_H_ 7.05 (d, *J* = 8.4 Hz, 2H, H-6′, H-8′) and 6.67 (d, *J* = 8.4 Hz, 2H, H-5′, H-9′), indicating a para-disubstituted benzene ring in **1**; and two active hydrogens at *δ*_H_ 9.26 (s, 1H, 7′-OH) and 8.37 (d, *J* = 6.8 Hz, 1H, 2′-NH). Twenty-six carbons, including four carbonyls (three ester carbonyls at *δ*_C_ 174.1, 173.1, and 171.3, and one amide carbonyl at *δ*_C_ 170.0), six aromatic carbons (*δ*_C_ 156.2, 130.2 × 2, 126.9, and 115.1 × 2), two olefinic carbons (*δ*_C_ 165.0 and 124.0), two quaternary carbons (*δ*_C_ 59.5 and 39.9), three methines (*δ*_C_ 75.2, 54.5, and 40.8), seven methylenes (*δ*_C_ 70.2, 67.9, 35.6, 29.5, 22.8, 19.8, and 18.9), and two methyls (*δ*_C_ 22.2 and 14.6), were displayed in the ^13^C NMR (Table 1). These functional groups represented nine degrees of unsaturation. Thus, the last four degrees of unsaturation indicated four additional rings in **1**. The characteristics of these NMR data suggested that **1** has substructures of a drimane-type sesquiterpene and an *N*-acetyl tyrosine [25, 26] (Fig. 1).

**Table 1:** ^1^H (400 MHz) and ^13^C NMR (100 MHz) Data (DMSO-*d*_6_) for **1** and **2**

	**1**		**2**	
	*δ*_C_	*δ*_H_ (*J* in Hz)	*δ*_C_	*δ*_H_ (*J* in Hz)
1	75.2	4.63, dd (10.8, 4.0)	71.1	3.49, ddd (10.2, 5.2, 3.8)
2a	22.8	1.81, d (4.0)	27.3	1.74, overlapped
2b		1.35, m		1.63, m
3a	29.5	2.09, overlapped	30.1	2.06, overlapped
3b		1.77, dd (13.8, 4.0)		1.74, overlapped
4	59.5		59.6	
5	40.8	1.98, overlapped	41.3	1.74, overlapped
6a	18.9	1.98, overlapped	19.1	1.97, m
6b		1.51, dd (10.6, 7.4)		1.52, m
7a	19.8	2.25, d (16.8)	19.9	2.25, dd (17.2, 5.0)
7b		2.09, overlapped		2.13, overlapped
8	124.0		123.0	
9	165.0		167.6	
10	39.9		41.5	
11a	70.2	4.92, dt (18.0, 2.2)	70.9	4.86, dd (5.0, 2.8)
11b		4.71, dd (18.0, 2.2)		
12	173.1		173.4	
13a	67.9	4.51, d (5.6)	68.1	4.49, d (5.6)
13b		4.30, d (5.6)		4.35, d (5.6)
14	174.1		174.7	
15	14.6	0.90, s	13.7	0.89, s
1′	171.3			
2′	54.5	4.35, dd (14.4, 7.6)		
3′	35.6	2.82, d (7.6)		
4′	126.9			
5′	130.2	6.67, d (8.4)		
6′	115.1	7.05, d (8.4)		
7′	156.2			
8′	115.1	7.05, d (8.4)		
9′	130.2	6.67, d (8.4)		
1′′	170.0			
2′′	22.2	1.83, s		
1-OH				5.18, d (5.2)
7′-OH		9.26, s		
NH		8.37, d (6.8)

**Fig. 2:**
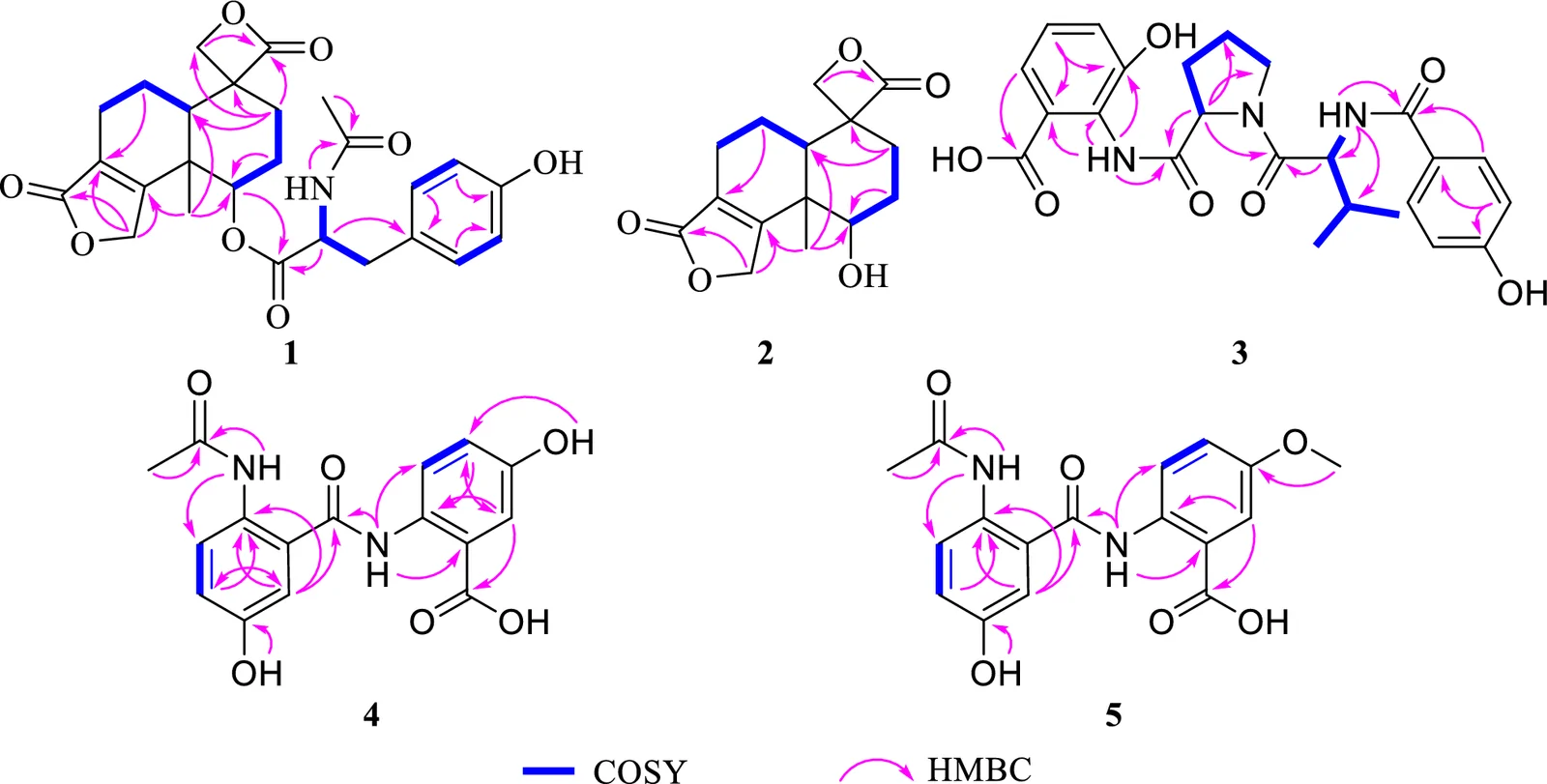
COSY and key HMBC correlations of **1**–**5**

The planar structure of **1** was established by analyses of 2D NMR spectra (Fig. 2). The ^1^H–^1^H COSY correlations of H-1/H-2/H-3 and H-5/H-6/H-7, coupled with the key HMBC correlations of H-3/C-5, H-6/C-8, H-15/C-1, C-5 and C-9, and H-11/C-8, C-9 and C-12, supported the existence of a drimane-type sesquiterpene fragment. The ^1^H–^1^H COSY cross-peaks of NH/H-2′/H-3′, H-5′/H-6′, and H-8′/H-9′, aided by HMBC correlations of H-2′/C-1′ and C-4′, NH/C-1′′, H-2′′/C-1′′, H-5′/C-4′, and H-6′/ C-7′, established the *N*-acetyl tyrosine moiety. The HMBC correlation of H-1/C-1′ unambiguously demonstrated the drimane-type sesquiterpene unit linked the *N*-acetyl tyrosine residue via an ester bond. Furthermore, the final degree of unsaturation, the oxygenated methylene C-13 (*δ*_C_ 67.9), and the ester carbonyl C-14 (*δ*_C_ 174.1) formed a spiro *β*-lactone fragment connected to C-4, as evidenced by the HMBC correlations of H-3/C-4, C-13 and C-14, and H-13/C-14. Consequently, the planar structure of **1** was established.

**Fig. 3:**
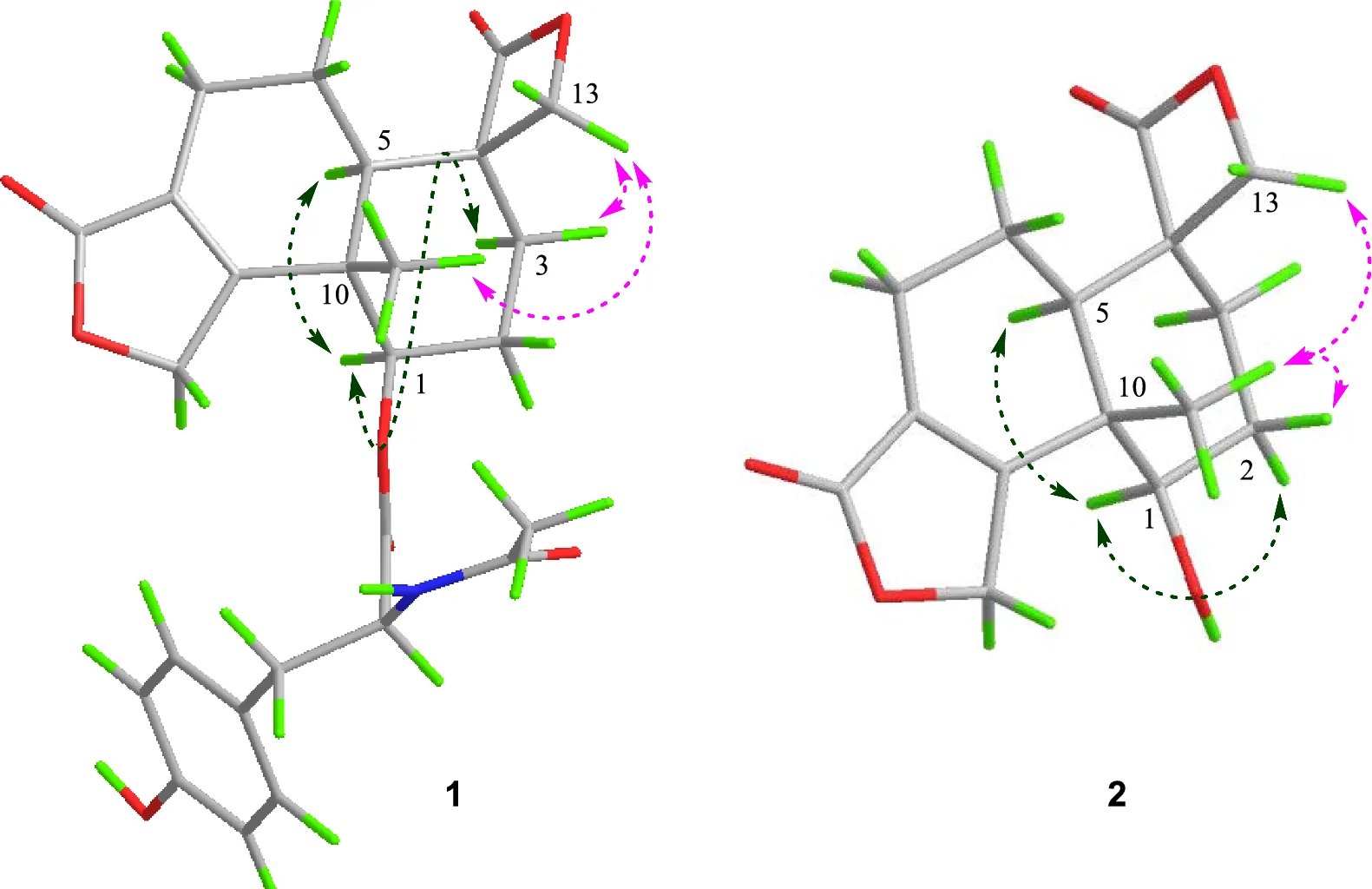
Key NOESY correlations of **1** and **2**

**Fig. 4:**
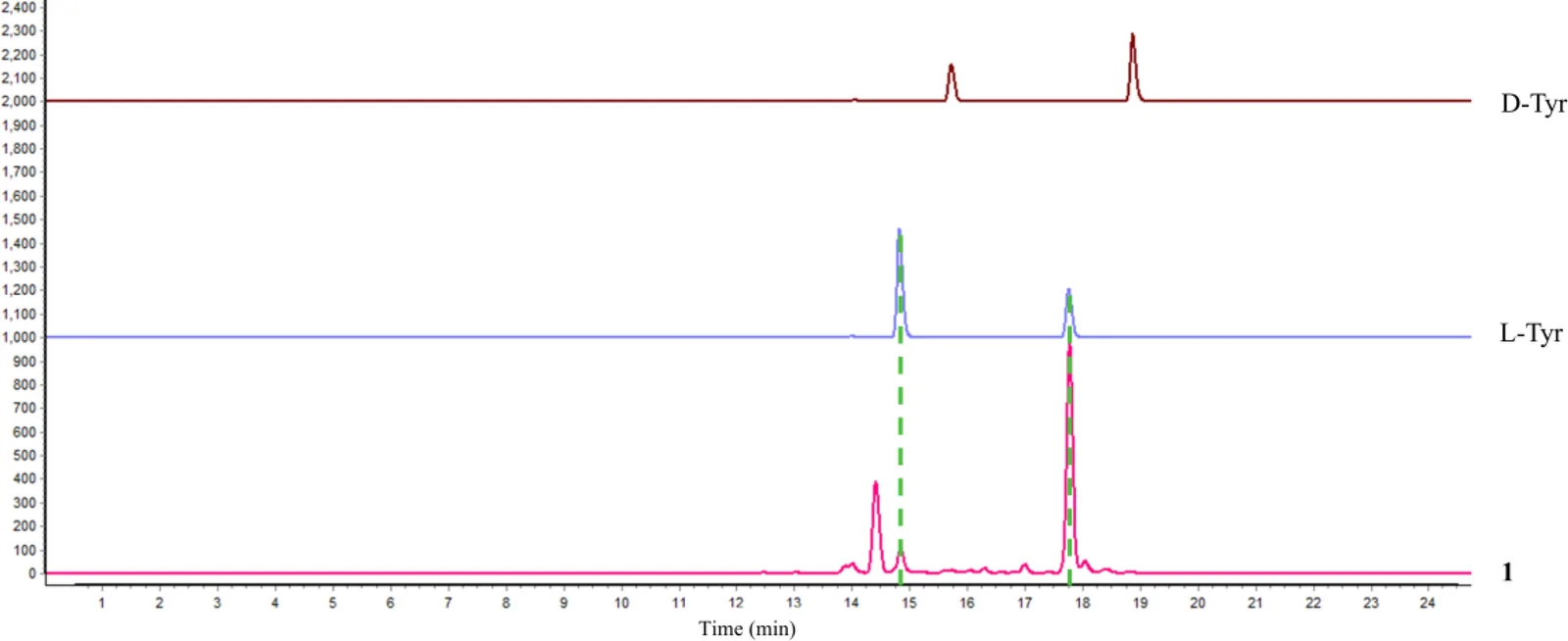
HPLC analyses of standard amino acids and hydrolysates of **1**

**Fig. 5:**
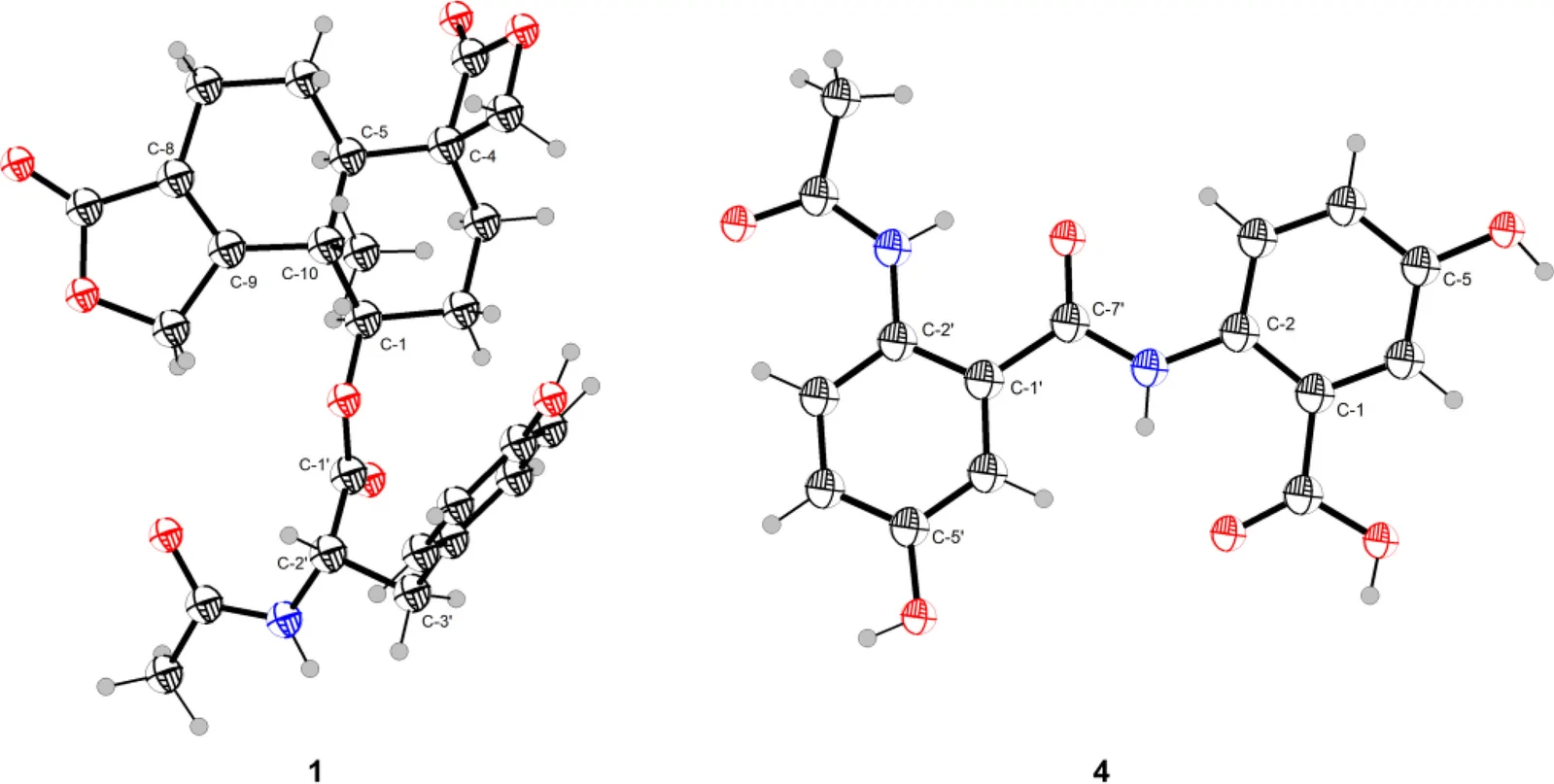
X-ray crystallographic structures of **1** and **4**

The relative configurations of C-1, C-4, C-5, and C-10 in the drimane-type sesquiterpene unit were elucidated by the NOESY experiment (Fig. 3). The NOESY cross-peaks of H-1/H-3α/H-5 suggested these protons were on the same orientation, whereas H-3β/H-13/H-15 indicated these protons to be another orientation. Marfey’s reagent (*L*-FDAA) derivatization [27] of the acid hydrolysate, followed by HPLC analysis (Fig. 4), established the absolute configuration of the amino acid residue. By comparison with the standard retention times, an *L*-Tyr residue was confirmed in **1**. The planar structure of **1** was further validated, and its absolute configuration was assigned as 1*R*,4*R*,5*R*,10*S*,2′*S* [Flack parameter –0.13 (10)] by X-ray diffraction (Fig. 5).

Compound **1** is a hybrid natural product consisting of a drimane-type sesquiterpene moiety and an *N*-acetyl tyrosine unit. Sesquiterpene-amino acid conjugates, a rare subclass of natural products, have emerged as promising bioactive metabolites owing to their wide-ranging pharmacological properties, e.g., anti-neuroinflammatory activity [28], antiviral activity [29], cytotoxicity [30], and so on. These compounds are usually obtained from plants [28–32], whereas a few are isolated from fungi [33, 34].

**Fig. 6:**
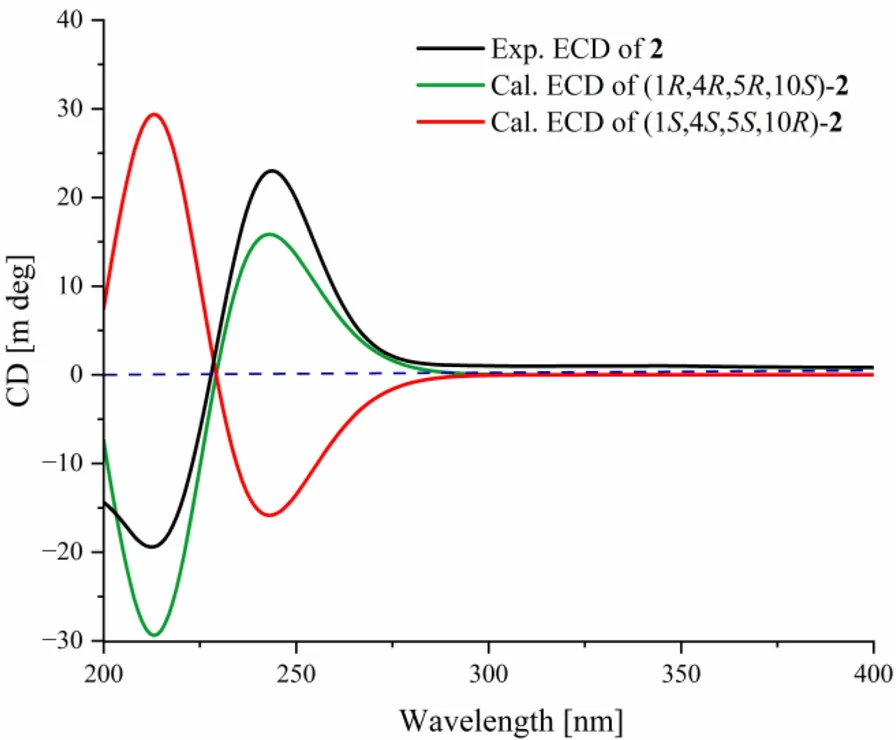
Experimental and calculated ECD spectra of **2**

Asperdrimane B (**2**) was assigned a molecular formula of C_15_H_18_O_5_ (Ω = 7) by HR-ESIMS (*m/z* 279.1226, calcd. for C_15_H_19_O_5_^+^). The ^1^H NMR spectrum (Table 1) displayed characteristic signals for a drimane-type sesquiterpene skeleton, including a methyl at *δ*_H_ 0.89 (s, 3H, H-15); six methylene signals at *δ*_H_ 4.86 (dd, *J* = 5.0, 2.8 Hz, 2H, H-11), 4.49 (d, *J* = 5.6 Hz, 1H, H-13a), 4.35 (d, *J* = 5.6 Hz, 1H, H-13b), 2.25 (dd, *J* = 17.2, 5.0 Hz, 1H, H-7a), 2.13 (overlapped, 1H, H-7b), 2.06 (overlapped, 1H, H-3a), 1.97 (m, 1H, H-6a), 1.74 (overlapped, 2H, H-2a, H-3b), 1.63 (m, 1H, H-2b), and 1.52 (m, 1H, H-6b); and two methines at *δ*_H_ 3.49 (ddd, *J* = 10.2, 5.2, 3.8 Hz, 1H, H-1) and 1.74 (overlapped, 1H, H-5). Two carbonyls (*δ*_C_ 174.7 and 173.4), two olefinic carbons (*δ*_C_ 167.6 and 123.0), two methine carbons (*δ*_C_ 71.1 and 41.3), six methylene carbons (*δ*_C_ 70.9, 68.1, 30.1, 27.3, 19.9, and 19.1), one methyl carbon (*δ*_C_ 13.7), and two quaternary carbons (*δ*_C_ 59.6 and 41.5) were observed in the ^13^C NMR (Table 1). Two carbonyl groups and one olefinic double bond accounted for three degrees of unsaturation, implying four rings in **2**. Overall, **2** showed NMR data similar to those of **1** but lacked the signals of the *N*-acetyl tyrosine residue, indicating that **2** was a drimane-type sesquiterpene. Comprehensive 2D NMR analyses (Fig. 2) helped to establish the planar structure of **2**. The NOESY correlations (Fig. 3) of H-1/H-2α/H-5 indicated H-1 and H-5 were on the same face, while the relationships of H-15/H-2β/H-13 revealed H-13 and H-15 on the opposite face. Ultimately, ECD spectroscopy and TDDFT calculations established the absolute configuration of** 2** as 1*R*,4*R*,5*R*,10*S* (Fig. 6).

The molecular formula of asperspamide A (**3**) was determined as C_24_H_27_N_3_O_7_ by HR-ESIMS at *m/z* 470.1924 [M + H]^+^ (calcd. for C_24_H_28_N_3_O_7_^+^, 470.1922). The ^1^H NMR spectra (Table 2) revealed two methyls at *δ*_H_ 0.95 (d, *J* = 6.6 Hz, 3H, H-4′′) and 0.92 (d, *J* = 6.6 Hz, 3H, H-5′′); three sets of methylenes at *δ*_H_ 3.94 (m, 1H, H-5′a), 3.69 (m, 1H, H-5′b), 2.06 (m, 2H, H-3′), 2.04 (m, 1H, H-4′a), 1.91 (m, 1H, H-4′b); three methines at *δ*_H_ 4.58 (t, *J* = 5.6 Hz, 1H, H-2′), 4.47 (t, *J* = 8.8 Hz, 1H, H-2′′), and 2.15 (m, 1H, H-3′′); and two amide (-NH) proton signals at *δ*_H_ 9.92 (s, 1H, 2-NH) and 8.22 (d, *J* = 8.0 Hz, 1H, 2′′-NH). Seven aromatic protons at *δ*_H_ 7.79 (d, *J* = 8.8 Hz, 2H, H-2′′′, H-6′′′), 7.24 (dd, *J* = 8.0, 1.8 Hz, 1H, H-6), 7.11 (t, *J* = 8.0 Hz, 1H, H-5), 7.06 (dd, *J* = 8.0, 1.8 Hz, 1H, H-4), and 6.79 (d, *J* = 8.8 Hz, 2H, H-3′′′, H-5′′′), indicated a para-disubstituted and a 1,2,3-trisubstituted benzene ring in **3**. The ^13^C NMR spectrum (Table 2) showed characteristics of an amide derivative, including four carbonyls (*δ*_C_ 171.0, 170.9, 168.3, and 166.2), twelve aromatic carbons (*δ*_C_ 160.4, 151.1, 129.7 × 2, 127.4, 125.8, 124.6 × 2, 120.9, 119.9, and 114.8 × 2), three methines [two of which are heteroatom-bonded (*δ*_C_ 60.2 and 56.8)], three methylenes [one of which is connected to heteroatom (*δ*_C_ 47.4)], and two methyls (*δ*_C_ 19.2 and 19.1). Four partial structures, 3-hydroxyanthranilic acid residue, proline residue, valine residue, and *p*-hydroxybenzoic acid residue, were deduced from the NMR spectra.

**Table 2:** ^1^H (400 MHz) and ^13^C NMR (100 MHz) Data (DMSO-*d*_6_) for **3**

	*δ*_C_	*δ*_H_ (*J* in Hz)		*δ*_C_	*δ*_H_ (*J* in Hz)
1	127.4		5′b		3.69, m
2	124.6		1′′	171.0	
3	151.1		2′′	56.8	4.47, t (8.8)
4	119.9	7.06, dd (8.0, 1.8)	3′′	29.2	2.15, m
5	125.8	7.11, t (8.0)	4′′	19.1	0.92, d (6.6)
6	120.9	7.24, dd (8.0, 1.8)	5′′	19.2	0.95, d (6.6)
7	168.3		1′′′	124.6	
1′	170.9		2′′′, 6′′′	129.7	7.79, d (8.8)
2′	60.2	4.58, t (5.6)	3′′′, 5′′′	114.8	6.79, d (8.8)
3′	28.6	2.06, m	4′′′	160.4	
4′a	24.6	2.04, m	7′′′	166.2	
4′b		1.91, m	2-NH		9.92, s
5′a	47.4	3.94, m	2′′-NH		8.22, d (8.0)

**Fig. 7:**
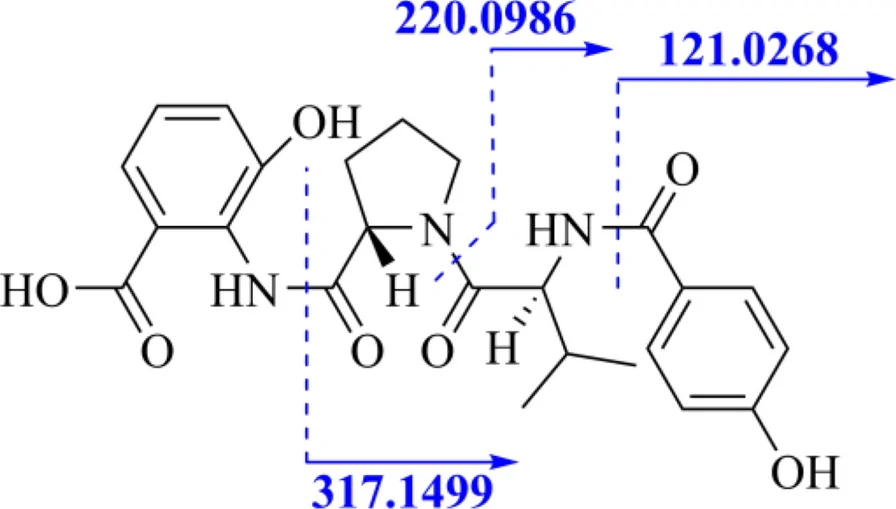
ESI-MS^2^ fragmentation of compound **3**

By analyzing inter-residue HMBC correlations, the connection order of amino acid fragments in **3** was determined. The HMBC correlations (Fig. 2) of 2-NH/C-1′, H-2′/C-1′′, and 2′′-NH/C-7′′′ allowed the definition of the residue sequence of **3** as 3-hydroxyanthranilic acid–proline–valine–*p*-hydroxybenzoic acid. The amino acid sequence was further confirmed by HR-ESIMS/MS fragmentation analysis with fragment ions at *m/z* 317.1499 [M–3-hydroxyanthranilic acid + H]^+^, 220.0986 [M–3-hydroxyanthranilic acid–proline + H]^+^, and 121.0268 [M–3-hydroxyanthranilic acid–proline–valine + H]^+^ (Fig. 7).

**Fig. 8:**
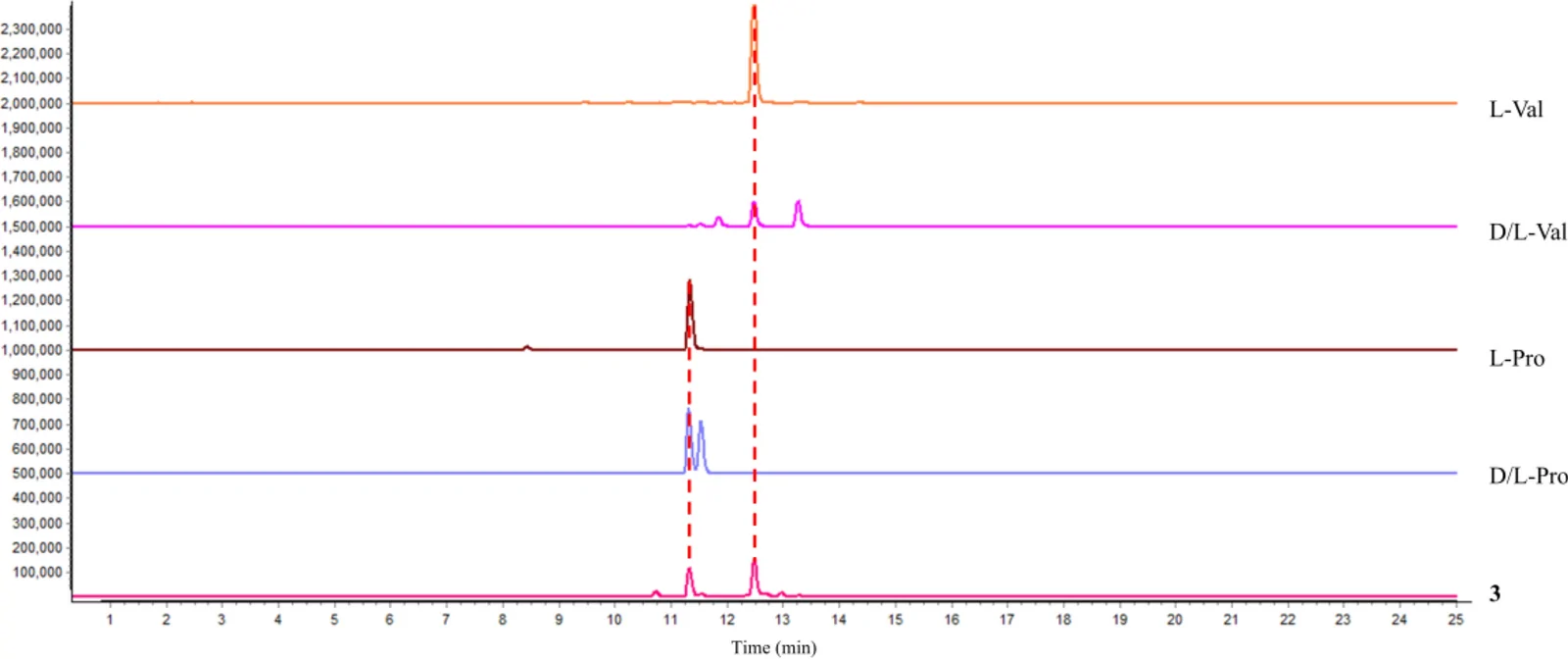
HPLC analyses of standard amino acids and hydrolysates of **3**

The chemical shifts of the *β*- and *γ*-carbon atoms (Δ*δ*_*βγ*_) of a given amino acid in the ^13^C NMR were diagnostic for the stereochemistry of the Pro amide bond. For *trans*-Pro, Δ*δ*_*βγ*_ typically falls between 0.0 and 4.8 ppm, whereas for *cis*-Pro, it ranges from 9.15 to 14.4 ppm [35]. The Δ*δ*_*βγ*_ value of 4.0 ppm for the proline residue in **3** supported its *trans* assignment. Marfey’s method enabled the assignment of the absolute configurations of the amino acid residues in **3** [27]. HPLC analysis of the FDAA derivative obtained from the hydrolysate of **3** revealed a retention time identical to that of the derivative synthesized from authentic *L*-Val and *L*-Pro (Fig. 8), confirming the *L*-configuration of both Val and Pro.

Asperspamide B (**4**) was isolated as colorless crystals with a molecular formula of C_16_H_14_N_2_O_6_ (*m*/*z* 331.0921, calcd. for C_16_H_15_N_2_O_6_^+^). A methyl at *δ*_H_ 1.97 (s, 3H), three active hydrogen signals at *δ*_H_ 9.66 (s, 2H), 10.05 (s, 1H), and 11.35 (s, 1H), and six aromatic protons at *δ*_H_ 8.36 (d, 1H, *J* = 9.0 Hz), 7.75 (d, 1H, *J* = 8.8 Hz), 7.40 (d, 1H, *J* = 3.0 Hz), 7.10 (d, 1H, *J* = 2.8 Hz), 7.06 (dd, 1H, *J* = 9.0, 3.0 Hz), and 6.92 (dd, 1H, *J* = 8.8, 2.8 Hz) were displayed in the ^1^H NMR (Table 3). Three carbonyls at *δ*_C_ 169.4, 168.0, and 165.6, twelve aromatic carbons at *δ*_C_ 153.7, 153.0, 132.5, 128.9, 127.4, 124.9, 122.6, 120.9, 119.0, 118.4, 116.7, and 114.1, and a methyl carbon at *δ*_C_ 24.0, were observed in the ^13^C NMR (Table 3). The ^1^H–^1^H COSY correlations (Fig. 2) of H-3/H-4 and H-3′/H-4′, along with the HMBC correlations of H-4/C-2, H-6/C-2 and C-4, H-4′/C-2′ and C-6′, and H-6′/C-2′ confirmed the existence of two 1,2,4-trisubstituted benzene rings. The HMBC correlations of 5-OH/C-4 and 5′-OH/C-5′ (Fig. 2) allowed the assignment of two hydroxyls at C-5 and C-5′. The HMBC correlation of H-6/C-7 placed a carboxyl at C-1. Additionally, an acetylamino at C-2′ was evidenced by the HMBC correlations of H-2′′/C-1′′, and 2′-NH/C-3′ and C-1′′. Another acetylamino group linked to C-2 was established by the HMBC correlations of 2-NH/C-3 and C-7′, and H-6′/C-7′. Lastly, the structure of **4** was further validated through its X-ray diffraction data (Fig. 5).

**Table 3:** ^1^H (400 MHz) and ^13^C NMR (100 MHz) Data (DMSO-*d*_6_) for **4** and **5**

	**4**		**5**	
	*δ*_C_	*δ*_H_ (*J* in Hz)	*δ*_C_	*δ*_H_ (*J* in Hz)
1	119.0		118.9	
2	132.5		133.9	
3	122.6	8.36, d (9.0)	122.5	8.47, d (9.2)
4	120.9	7.06, dd (9.0, 3.0)	120.1	7.26, dd (9.2, 3.0)
5	153.0		154.6	
6	116.7	7.40, d (3.0)	114.9	7.48, d (3.0)
7	169.4		169.2	
1′	127.4		128.7	
2′	128.9		127.6	
3′	124.9	7.75, d (8.8)	125.1	7.71, d (8.8)
4′	118.4	6.92, dd (8.8, 2.8)	118.5	6.92, dd (8.8, 2.8)
5′	153.7		153.7	
6′	114.1	7.10, d (2.8)	114.1	7.10, d (2.8)
7′	165.6		165.8	
1′′	168.0		168.0	
2′′	24.0	1.97, s	23.9	1.97, s
5-OCH_3_			55.5	3.79, s
5-OH		9.66, s		
5′-OH		9.66, s		9.69
2-NH		11.35, s		11.40, s
2′-NH		10.05, s		10.01, s

The HR-ESIMS data of asperspamide C (**5**) revealed that its molecular formula was C_17_H_16_N_2_O_6_ (*m*/*z* 345.1089, calcd. for C_17_H_17_N_2_O_6_^+^). Compared with the NMR data of **4**, the difference was that 5-OH in** 4** was replaced by a methoxy group (*δ*_H_ 3.79, *δ*_C_ 55.5) in **5** (Table 3), which was evidenced by the HMBC correlation 5-OCH_3_/C-5 (Fig. 2). Accordingly, the structure of **5** was determined (Fig. 1).

### Antioxidant activity of compounds 1–9

**Fig. 9:**
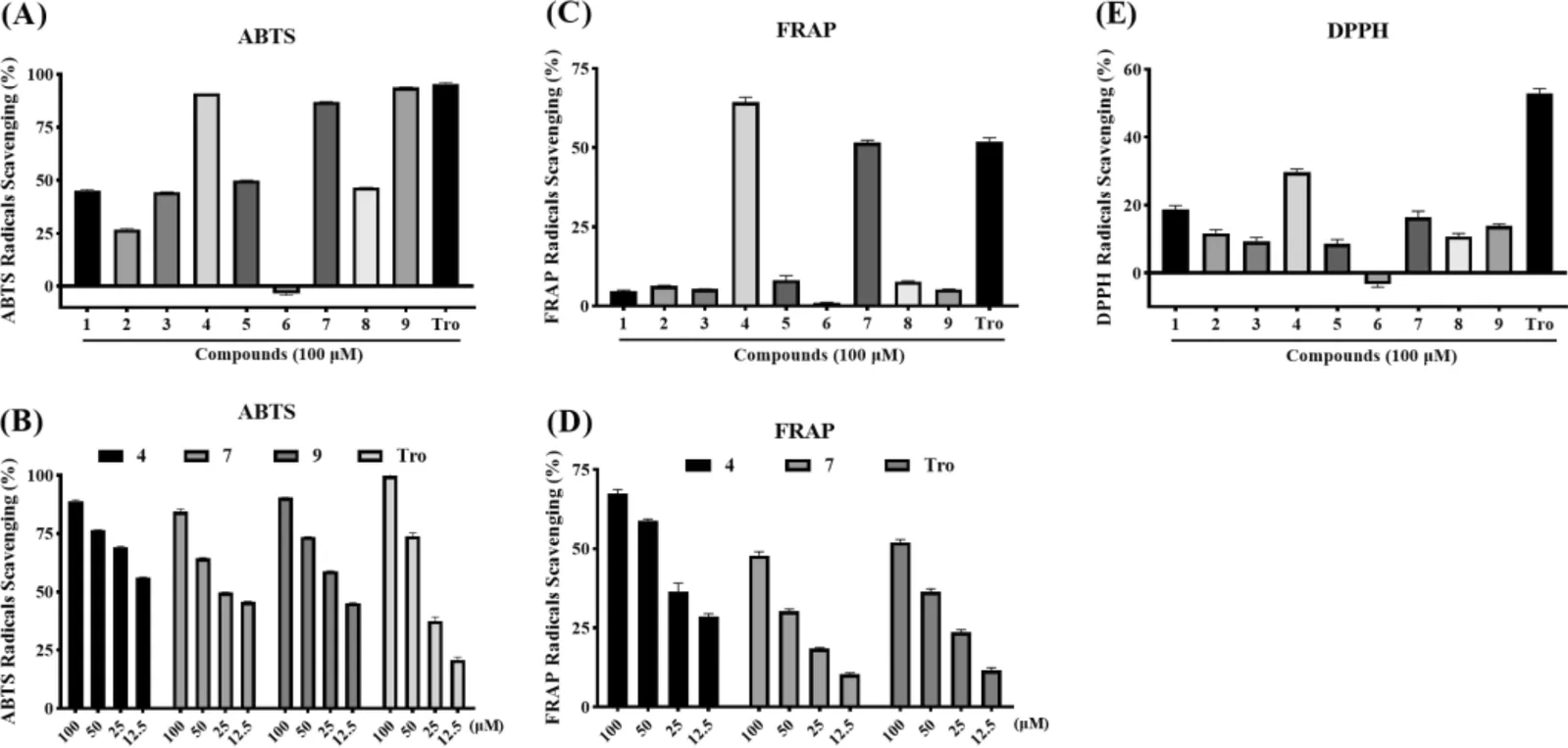
Antioxidant activities of **1–9**. **A** ABTS scavenging of **1**–**9** and Trolox; **B** ABTS scavenging of **4**, **7**, **9**, and Trolox; **C** FRAP scavenging of **1**–**9** and Trolox; **D** FRAP scavenging of **4**, **7**, and Trolox; **E** DPPH scavenging of **1**–**9** and Trolox

The ABTS, FRAP and DPPH assays were employed to assess the antioxidant capability of **1**–**9**. Compounds **4**, **7**, and **9** showed a significant ability to remove ABTS free radicals at 100 μM (Fig. 9A). Serial dilution from 100 μM further verified that these three compounds scavenge ABTS free radicals (Fig. 9B). They showed greater activity than the positive control Trolox when the concentration was below 50 μM. Especially, **4** showed particularly outstanding performance at all concentrations (12.5, 25, 50 and 100 μM). The FRAP test indicated that **4** and **7** exhibited strong antioxidant activity (Fig. 9C). And then, the subsequent activity verification was carried out with a gradient dilution from 100 to 12.5 μM. As shown in Fig. 9D, the activity of compound **4** was better than that of Trolox at any concentration in FRAP free radicals scavenging. Unfortunately, at 100 μM, all compounds showed weaker DPPH radical scavenging activity than Trolox (Fig. 9E).

### Cytotoxicity *and* in vitro *neuroprotective activity of compounds****1****–****9***

**Fig. 10:**
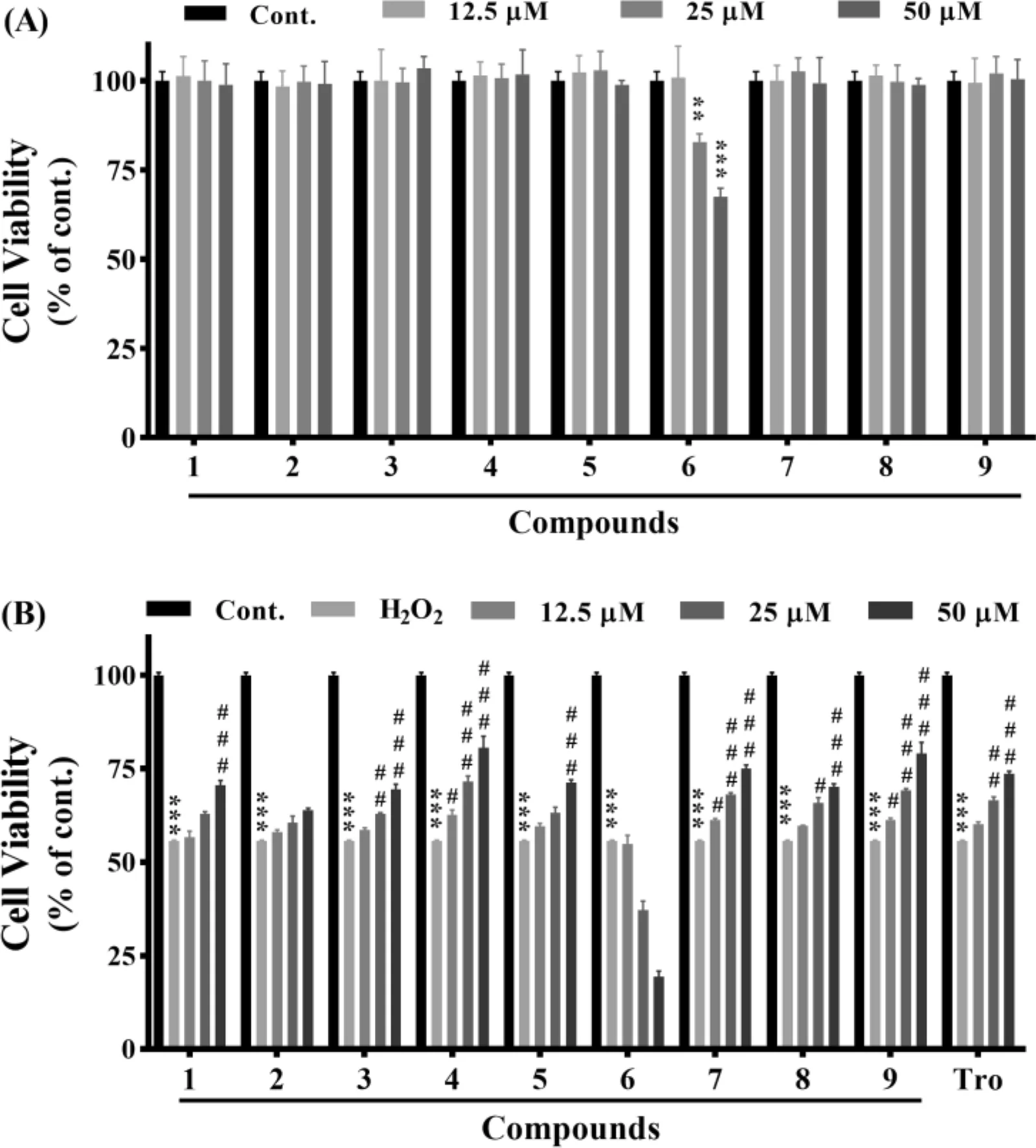
Cytotoxicity (**A**) and neuroprotection (**B**) of **1**–**9**. Statistical analysis showed that: ^***^ *P* < 0.001, compared with the control group; ^#^ *P* < 0.05, ^##^ *P* < 0.01, ^###^ *P* < 0.001, compared with the H_2_O_2_ treated group

The CCK-8 reagent was employed to assess both the cytotoxicity and the neuroprotective activity of **1**–**9** against H_2_O_2_-induced PC12 cell damage. No significant loss of PC12 cell viability was observed after treatment with **1**–**9** at concentrations below 50 μM relative to the blank control, except for **6** (Fig. 10A). Exposure to 60 μM H_2_O_2_ reduced PC12 cell viability by approximately 50%. Compounds **4**, **7**, and **9** markedly enhance cell viability at all tested concentrations (12.5, 25, and 50 μM); **3** and **8** presented protective effects at both 25 and 50 μM; **1** and **5** restored the cell viability only at 50 μM, which was the same as Trolox; **2** had no protective effect on H_2_O_2_ damaged PC12 cells, while **6** aggravated the cell damage at 25 and 50 μM (Fig. 10B).

### Inhibitory activity of compound 4 on intracellular ROS level

**Fig. 11:**
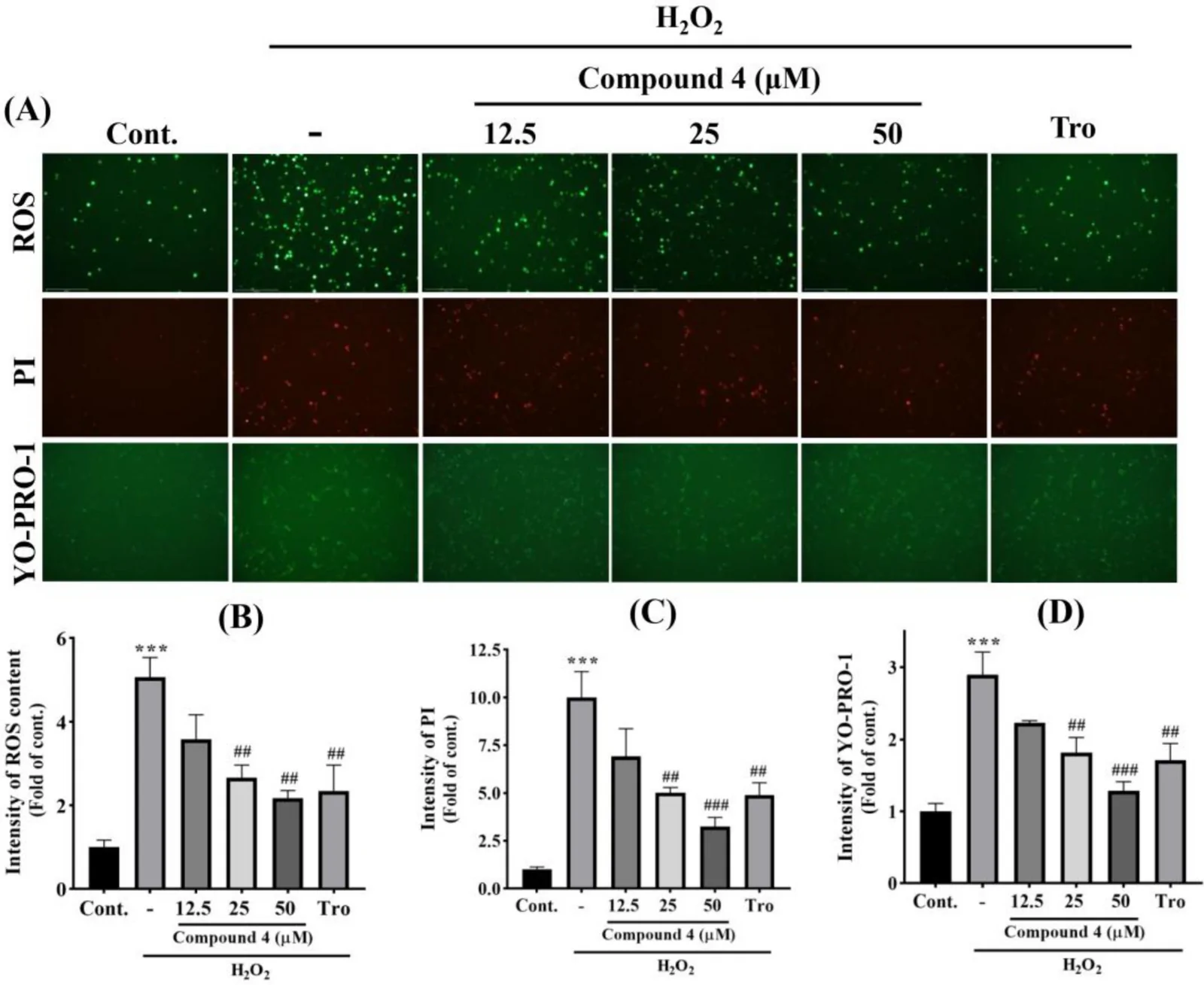
The neuroprotective effect of **4**. **A** and **B** The ROS content in H_2_O_2_-induced PC12 cells was inhibited by **4**, scale bar = 200 μm; **A**, **C** and **D 4** reduced H_2_O_2_-induced apoptosis/necrosis stained by YO-PRO-1 (green) and PI (red), scale bar = 200 μm. ^***^ *P* < 0.001, compared with the normal control group; ^#^ *P* < 0.05, ^##^ *P* < 0.01, ^###^ *P* < 0.001, compared with the H_2_O_2_ injury group

The above results indicated that compound **4** was worth further investigation of its neuroprotective effect. Increased ROS production is a prominent hallmark of the in vitro oxidative stress model established by exposing PC12 cells to H_2_O_2_. DCFH-DA, a fluorescent probe, was applied to stain PC12 cells that generated ROS in each group. After being damaged by H_2_O_2_, the level of ROS in PC12 cells was significantly increased, and bright green fluorescence was visible via the GFP channel of the EVOS™ FL Auto 2 imaging system (Fig. 11A). Notably, **4** reduced ROS levels in a concentration-dependent manner. Furthermore, at 50 μM, **4** showed better ROS scavenging activity than the positive control Trolox (Fig. 11B).

### Anti-apoptosis and necrosis activity of compound 4

Apoptotic cells are stained by YO-PRO-1 (YO), while necrotic cells are stained by propidium iodide (PI). These two dyes were used to evaluate whether compound **4** protects PC12 cells from H_2_O_2_-induced apoptosis and necrosis. After stained by green fluorescence (YO, marked apoptotic cells) and red fluorescence (PI, marked necrotic cells) dyes, a significant fluorescence signal could be observed in Fig. 11A. When compound **4** presented, apoptotic and necrotic cell numbers fell considerably in a concentration-dependent fashion. In addition, at 50 μM, **4** showed a significantly stronger protective effect on PC12 cells than the positive control Trolox (Figs. 11C and D). The above results fully demonstrated that compound **4** exhibited significant neuroprotective activity.

## Conclusion

In summary, five undescribed compounds, including two drimane-type sesquiterpenoids and three amides, together with four known compounds, were isolated from the fungus *Aspergillus spelaeus* GDGJ-286. Asperdrimane A (**1**) is the first heterodimer between drimane-type sesquiterpene and *N*-acetyl tyrosine units found from the natural resources. Asperspamide B (**4**) and WIN 66306 (**7**) exhibited outstanding antioxidant capacity in ABTS, FRAP and DPPH tests. Asperspamide B (**4**), WIN 66306 (**7**), and nectriatide (**9**) effectively protected PC12 cells from oxidative injury induced by H_2_O_2_. In particular, asperspamide B (**4**) was found to have strong anti-apoptosis and anti-necrosis ability. This active compound is expected to serve as a lead for antioxidant agents.

## Experimental

### General procedures

UV, IR, NMR, HRESIMS, and LC–MS/MS data were recorded using standard instruments. Melting points and optical rotations were measured conventionally. Crystal data were acquired on a diffractometer. Purification was carried out by column chromatography (silica gel, ODS) and HPLC.

### Fungal material, fermentation, and extraction

The strain GDGJ-286 (GenBank PP217298) was taxonomically classified as *Aspergillus spelaeus*. The methodologies employed for fermentation and extraction have been previously documented [21].

### Isolation

Silica gel column chromatography of the EtOAc extract under increasing polarity gradient afforded six fractions (Fr.1–Fr.6). Further fractionation of Fr.3 by ODS chromatography with gradient MeOH–H_2_O gave six subfractions, from which **6** (51.0 mg) was obtained via recrystallization of the third subfraction. Fr.4 was subjected to ODS under similar gradient elution to afford eight subfractions. The second subfraction was purified by successive silica gel column chromatography and HPLC (MeOH-H_2_O, 40:60, *t*_R_ = 14.8 min), yielding **2** (29.5 mg), whereas **5** (20.5 mg) was isolated from the fourth by recrystallization. Similarly, Fr.5 was fractionated on ODS with gradient MeOH-H_2_O to yield six subfractions. Compound** 4** (31.5 mg) was crystallized from the first subfraction, while **1** (55.8 mg, *t*_R_ = 15.6 min) and **9** (16.2 mg, *t*_R_ = 24.2 min) were purified from the second subfraction by ODS and HPLC (MeOH-H_2_O, 50:50). Compound** 3** (52.7 mg) was obtained from the third subfraction via HPLC (MeOH-H_2_O, 50:50, *t*_R_ = 22.4 min) following ODS separation. Finally, Fr.6 was separated on ODS with gradient MeOH-H_2_O to produce four subfractions, from which **8** (12.3 mg) and **7** (78.4 mg) were isolated by HPLC (MeCN-H_2_O, 55:45, *t*_R_ = 23.8 min) and (MeCN-H_2_O, 40:60, *t*_R_ = 21.2 min), respectively.

Asperdrimane A (**1**): colorless crystals; m.p. 220.4–221.6 °C; [*α*]$_{D}^{25}$ + 35.3 (*c* 0.1, MeOH); ECD (MeOH) *λ*_max_ (Δ*ε*) 212 (− 1.83), 235 (20.12) nm; UV (MeOH) *λ*_max_ ((log *ε*) 221 (3.1), 276 (0.6) nm; IR (KBr) *ν*_max_ 3510, 3349, 2919, 2850, 1800, 1742, 1668, 1500, 1442, 1380, 1195, 994, 890, 835 cm^−1^; ^1^H and ^13^C NMR data are listed in Table 1; HRESIMS *m*/*z* 484.1962 [M + H]^+^, theoretical value 484.1966 for C_26_H_30_NO_8_^+^.

Asperdrimane B (**2**): colorless oil; [*α*]$_{D}^{25}$  + 147.0 (*c* 0.1, MeOH); ECD (MeOH) *λ*_max_ (Δ*ε*) 209 (− 19.41), 243 (22.97) nm; UV(CH_3_OH) λ_max_ (log *ε*) 222 (3.2), 275 (0.2) nm; IR(KBr) *υ*_max_ 3443, 2920, 2852, 2350, 2322, 1818, 1740, 1662, 1440, 1384, 1073, 975, 886, 667 cm^−1^; ^1^H and ^13^C NMR data are listed in Table 1; HRESIMS *m*/*z* 279.1226 [M + H]^+^, theoretical value 279.1227 for C_15_H_19_O_5_^+^.

Asperspamide A (**3**): colorless oil; [*α*]$_{D}^{25}$ + 16.0 (*c* 0.1, MeOH); UV(CH_3_OH) λ_max_ (log *ε*) 200 (4.42), 274 (3.83), and 310 (3.70) nm; IR(KBr) *υ*_max_ 3407, 2935, 1712, 1685, 1608, 1468, 1275, 1164, 848 cm^−1^; ^1^H and ^13^C NMR data are listed in Table 2; HRESIMS *m*/*z* 470.1924 [M + H]^+^, theoretical value 470.1922 for C_24_H_28_N_3_O_7_^+^.

Asperspamide B (**4**): colorless crystals; m.p. 228.6–230.1 °C; [*α*]$_{D}^{25}$ + 2.9 (*c* 0.1, MeOH); UV(CH_3_OH) λ_max_ (log *ε*) 225 (3.6), 267 (2.0), 334 (1.7) nm; IR(KBr) *υ*_max_ 3269, 2923, 2801, 2692, 2605, 1660, 1604, 1535, 1440, 1286, 1223, 971, 879, 749 cm^−1^; ^1^H and ^13^C NMR data are listed in Table 3; HRESIMS *m*/*z* 331.0921 [M + H]^+^, theoretical value 331.0925 for C_16_H_15_N_2_O_6_^+^.

Asperspamide C (**5**): colorless crystals; m.p. 240.0–241.6 °C; [*α*]$_{D}^{25}$ + 2.9 (*c* 0.1, MeOH); UV(CH_3_OH) λ_max_ (log *ε*) 225 (3.6), 272 (2.2), 335 (1.7) nm; IR(KBr) *υ*_max_ 3413, 2938, 2826, 2623, 1678, 1607, 1539, 1490, 1287, 1219, 974, 830, 738 cm^−1^; ^1^H and ^13^C NMR data are listed in Table 3; HRESIMS *m*/*z* 345.1089 [M + H]^+^ theoretical value 345.1081 for C_17_H_17_N_2_O_6_^+^.

### Crystallographic characterization of 1 and 4

Colorless crystals of **1** and **4** were obtained from MeOH. Crystallographic data were deposited at the CCDC under deposition numbers 2479464 and 2479457.

*Crystal data for* **1** *cocrystallized with two molecules of methanol*: C_28_H_37_NO_10_, *M*r = 547.58, orthorhombic, space group P2_1_2_1_2_1_ with *a* = 10.58920(10) Å, *b* = 11.81890(10) Å, *c* = 22.2075(3) Å, *α* = *γ* = *β* = 90°, *V* = 2779.33(5) Å^3^, *D*x = 1.309 mg/m^3^, and *μ* (Cu K*α*) = 0.828 mm^−1^. The final *R*_1_ value was 0.0361, Flack parameter = –0.13 (10).

*Crystal data for* **4** *cocrystallized with two molecules of methanol*: C_18_H_22_N_2_O_8_, *M*r = 394.37, Triclinic, space group *P*-1 with *a* = 8.09110(10) Å, *b* = 11.1114(2) Å, *c* = 11.1400(2) Å, *α* = 83.2370(10)°, *β* = 71.5170(10)°, *γ* = 86.0850(10)°, *V* = 742.75(3) Å^3^, *D*x = 1.389 mg/m^3^, and *μ* (Cu K*α*) = 0.936 mm^−1^. The final *R*_1_ value was 0.0496.

### Marfey’s analysis of asperspamide A (3).

Asperspamide A (**3**, 0.5 mg) was subjected to acid hydrolysis and Marfey’s derivatization [36]. Standard amino acids (*L*-Val, *DL*-Val, *L*-Pro, *DL*-Pro) were treated using the same procedure. All FDAA derivatives were analyzed by HPLC (Shim-pack GIST C18, 4.6 × 150 mm, 5 μm, 1.0 mL/min) with 10–100% CH_3_CN in H_2_O for 0 − 17 min, 100% CH_3_CN for 17 − 24 min, and 10% CH_3_CN for 24 − 25 min.

### ECD calculation and ECD spectra acquisition

ECD and theoretical calculations for asperdrimane B (**2**) were performed following the reported method [19], with Gaussian 09 and Spartan’14 employed for calculations and initial conformation generation, respectively; conformations were optimized at B3LYP/6—31G(d), ECD calculations were conducted via TDDFT at wB97xd/6—311G(d,p), and spectra were processed with 0.3 eV sigma/gamma value and + 15 nm shift to match experimental results.

### Antioxidant and neuroprotective activity assays.

All assays were performed according to a previously reported method [37]. ABTS, FRAP and DPPH radical scavenging activities were determined using corresponding commercial kits; samples, Trolox (positive control) and blank were incubated in 96-well plates. PC12 cells were cultured in RPMI-1640 medium, and H_2_O_2_-induced oxidative injury was established. Neuroprotection and cytotoxicity were evaluated by CCK-8 assay, intracellular ROS by DCFH-DA staining, and apoptosis/necrosis by YO-PRO-1/PI double staining; fluorescence intensity was analyzed using ImageJ.

## Supplementary Information

Additional file 1 (PDF 2645 KB)

## Data Availability

All data generated or analyzed during this study are included in this published article and its supplementary information files.
