# Supplementary material for: Antioxidant metabolites from the endophytic fungus Aspergillus spelaeus
Source: Nat Prod Bioprospect. 2026 Nov 2;16(1):97. doi: 10.1007/s13659-026-00637-9 (PMC13728077; doi:10.1007/s13659-026-00637-9)
Supplement: Supplementary file 1 [file 13659_2026_637_MOESM1_ESM.pdf]

SUPPORTING INFORMATION

Antioxidant metabolites from the endophytic fungus *Aspergillus spelaeus*

Jia-Tong Zhou [#], Min Wang [#], Qian Wu, Min Liang, Wei-Feng Xu*, Jun Li, Li-Jun Zhang*, Rui-Yun Yang*

Key Laboratory for Chemistry and Molecular Engineering of Medicinal Resources (Ministry of Education of China), Guangxi Key Laboratory of Chemistry and Molecular Engineering of Medicinal Resources, University Engineering Research Center for Chemistry of Characteristic Medicinal Resources (Guangxi), School of Chemistry and Pharmaceutical Sciences, Guangxi Normal University, Guilin 541004, People’s Republic of China

[#] Jia-Tong Zhou and Min Wang contributed equally to this work.

Corresponding Authors

*E-mail: yang_rui_yun@163.com (R.-Y. Yang), ajun840618@mailbox.gxnu.edu.cn (L.-J. Zhang), xuweifeng_u@163.com (W.-F. Xu).

List of Supporting Information

Figure S1. HRESIMS of compound **1**··········4
Figure S2. ^{1}H NMR spectrum of **1** in DMSO-d_6··········4
Figure S3. ^{13}C NMR spectrum of **1** in DMSO-d_6··········5
Figure S4. ^{1}H-^{1}H COSY spectrum of **1** in DMSO-d_6··········5
Figure S5. HSQC spectrum of **1** in DMSO-d_6··········6
Figure S6. HMBC spectrum of **1** in DMSO-d_6 ··········6
Figure S7. NOESY spectrum of **1** in DMSO-d_6 ··········7
Figure S8. HRESIMS of compound **2**··········7
Figure S9. ^{1}H NMR spectrum of **2** in DMSO-d_6 ··········8
Figure S10. ^{13}C NMR spectrum of **2** in DMSO-d_6··········8
Figure S11. ^{1}H-^{1}H COSY spectrum of 2 in DMSO-d_6··········9
Figure S12. HSQC spectrum of **2** in DMSO-d_6··········9
Figure S13. HMBC spectrum of **2** in DMSO-d_6 ··········10
Figure S14. NOESY spectrum of **2** in DMSO-d_6 ··········10
Figure S15. HRESIMS of compound **3**··········11
Figure S16. ^{1}H NMR spectrum of **3** in DMSO-d_6 ··········11
Figure S17. ^{13}C NMR spectrum of **3** in DMSO-d_6··········12
Figure S18. ^{1}H-^{1}H COSY spectrum of **3** in DMSO-d_6··········12
Figure S19. HSQC spectrum of **3** in DMSO-d_6··········13
Figure S20. HMBC spectrum of **3** in DMSO-d_6··········13
Figure S21. ESI-MS^{2} fragmentation of compound **3**··········14
Figure S22. HRESIMS of compound **4**··········14
Figure S23. 1H NMR spectrum of **4** in DMSO-d_6··········15
Figure S24. ^{13}C NMR spectrum of **4** in DMSO-d_6··········15
Figure S25. ^{1}H-^{1}H COSY spectrum of **4** in DMSO-d_6··········16
Figure S26. HSQC spectrum of **4** in DMSO-d_6··········16
Figure S27. HMBC spectrum of **4** in DMSO-d_6··········17

Figure S28. HRESIMS of compound **5**······17
Figure S29. ^{1}H NMR spectrum of **5** in DMSO-d_6 ······18
Figure S30. ^{13}C NMR spectrum of **5** in DMSO-d_6······18
Figure S31. ^{1}H-^{1}H COSY spectrum of **5** in DMSO-d_6······19
Figure S32. HSQC spectrum of **5** in DMSO-d_6······19
Figure S33. HMBC spectrum of **5** in DMSO-d_6······20
Table S1. Crystal data and structure refinement for **1**······21
Table S2. Crystal data and structure refinement for **4**······22
Table S3. Important thermodynamic parameters and Boltzmann distributions of the optimized **2** at B3LYP/6-31G(d) level in gas phase······23
Table S4. The coordinates for the lowest energy conformers of **2**······23

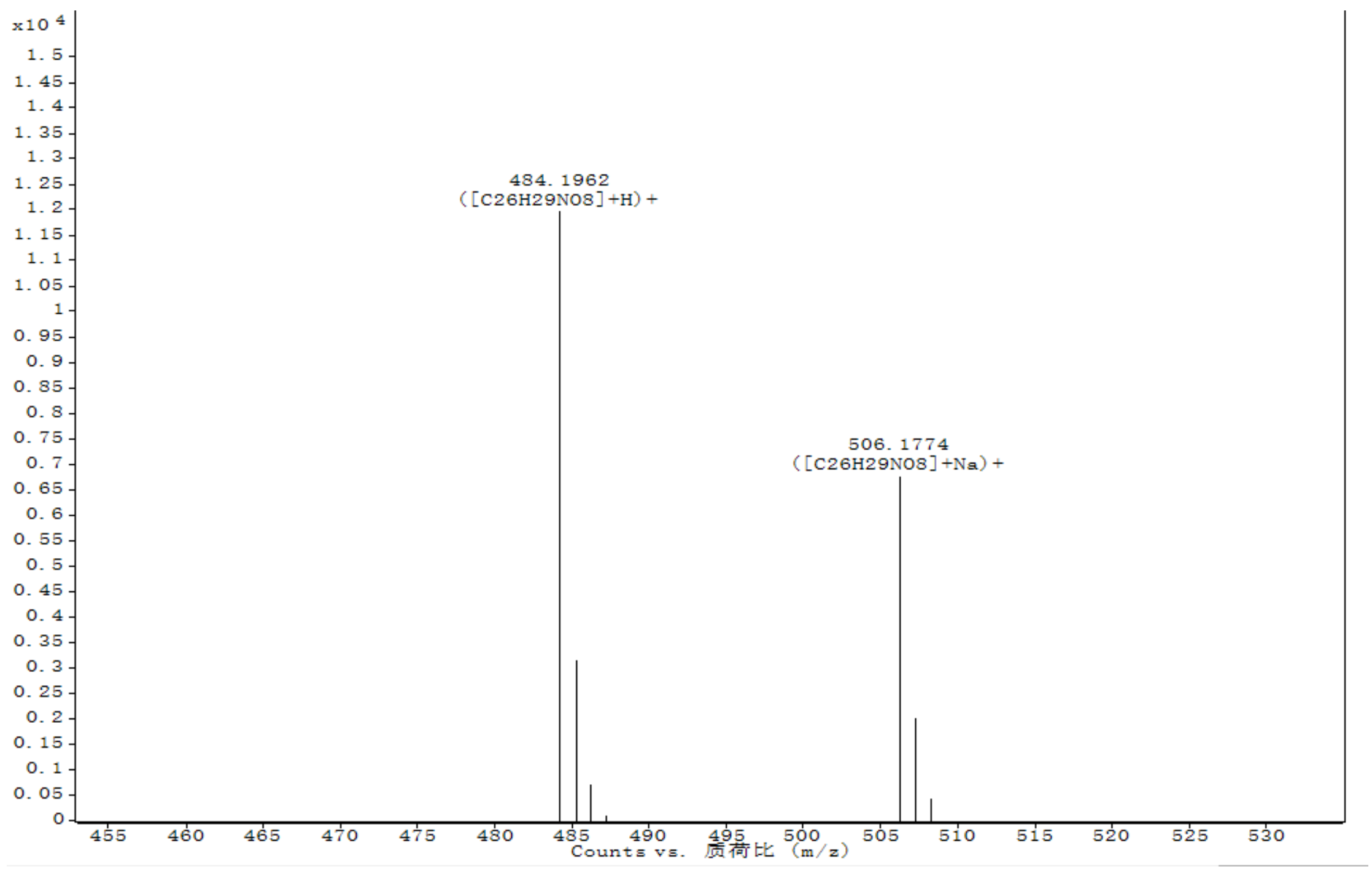

Figure S1. HRESIMS of compound **1**

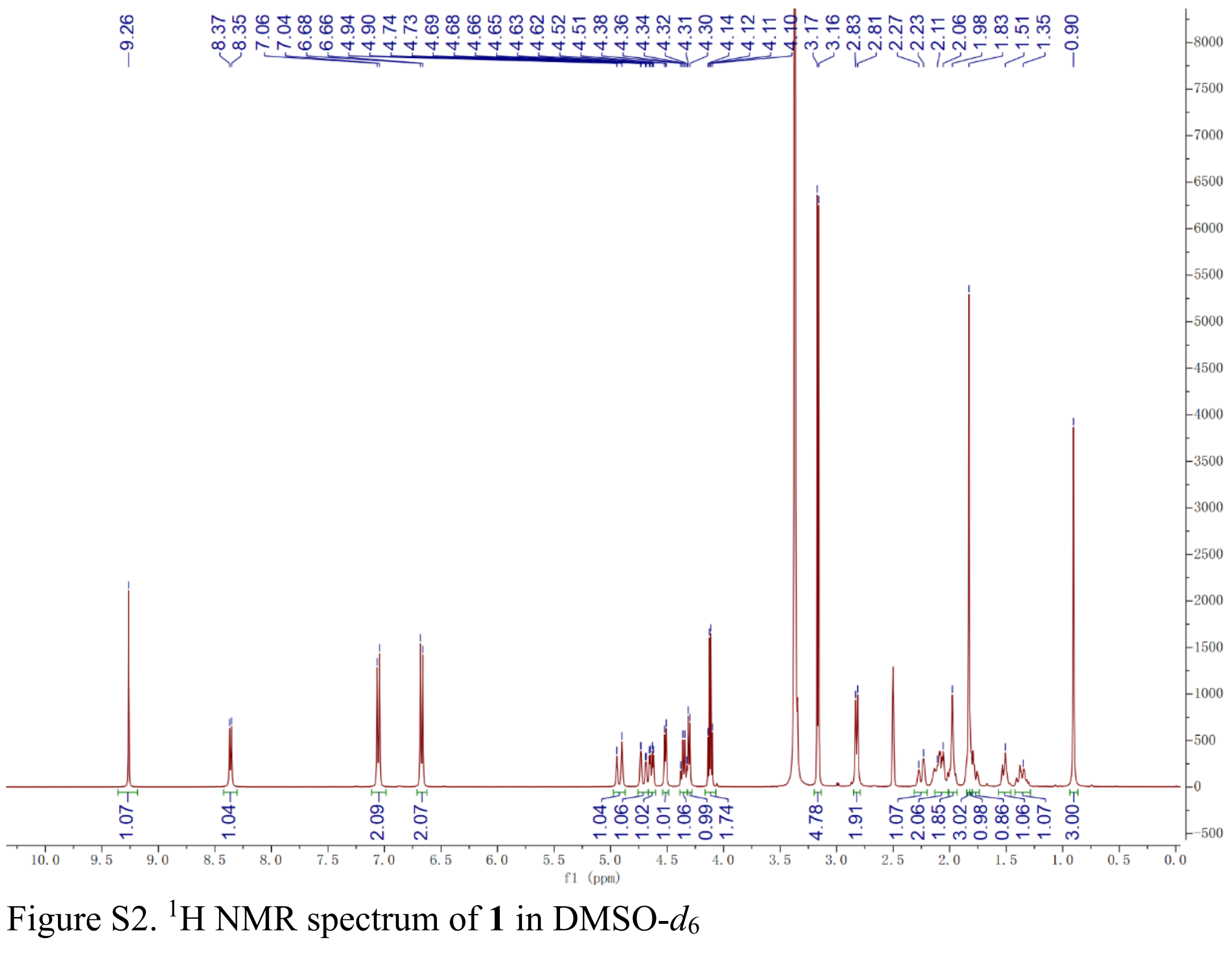

Figure S2. ^{1}H NMR spectrum of **1** in DMSO-d_6

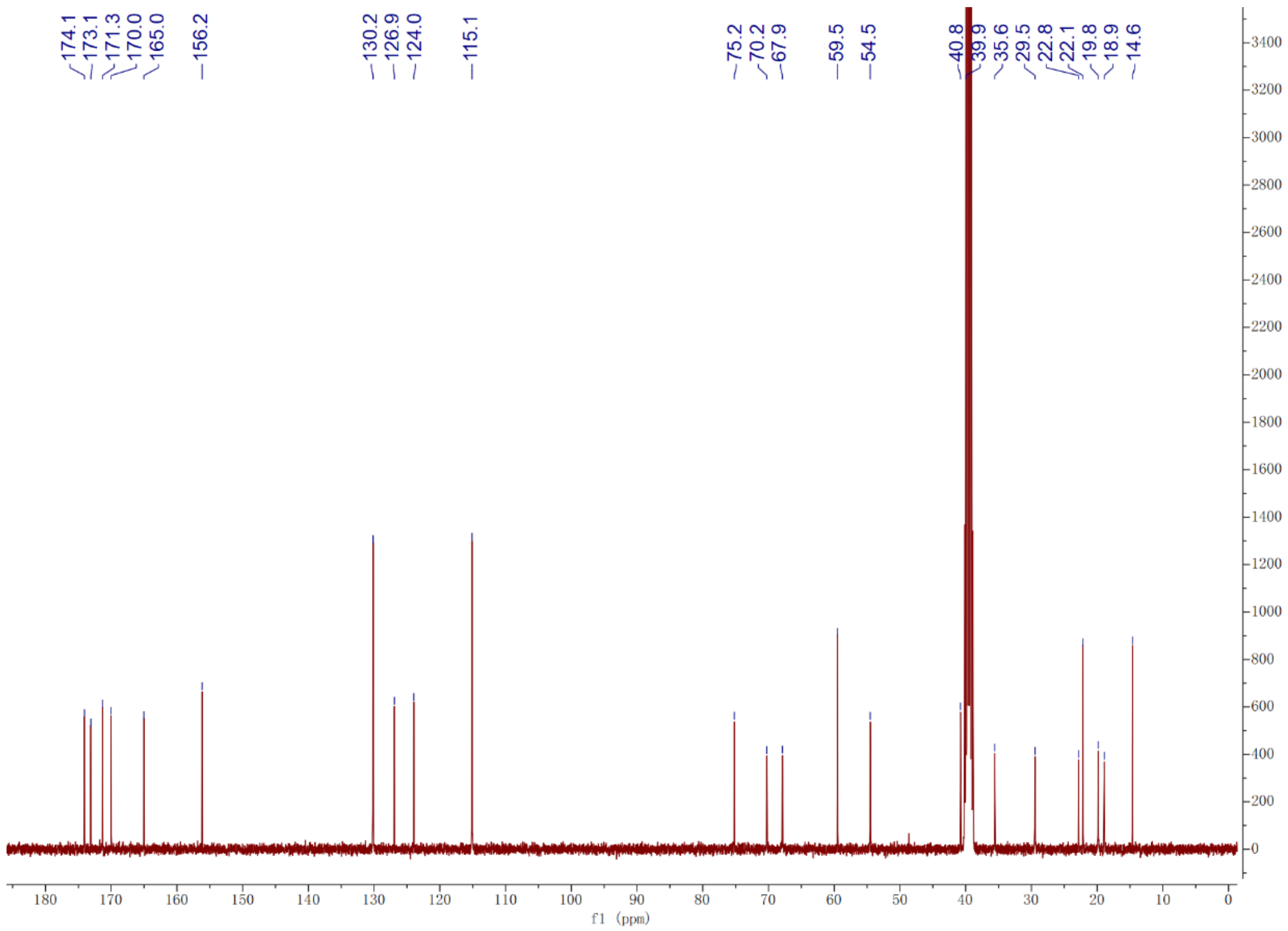

Figure S3. ^{13}C NMR spectrum of **1** in DMSO-d_6

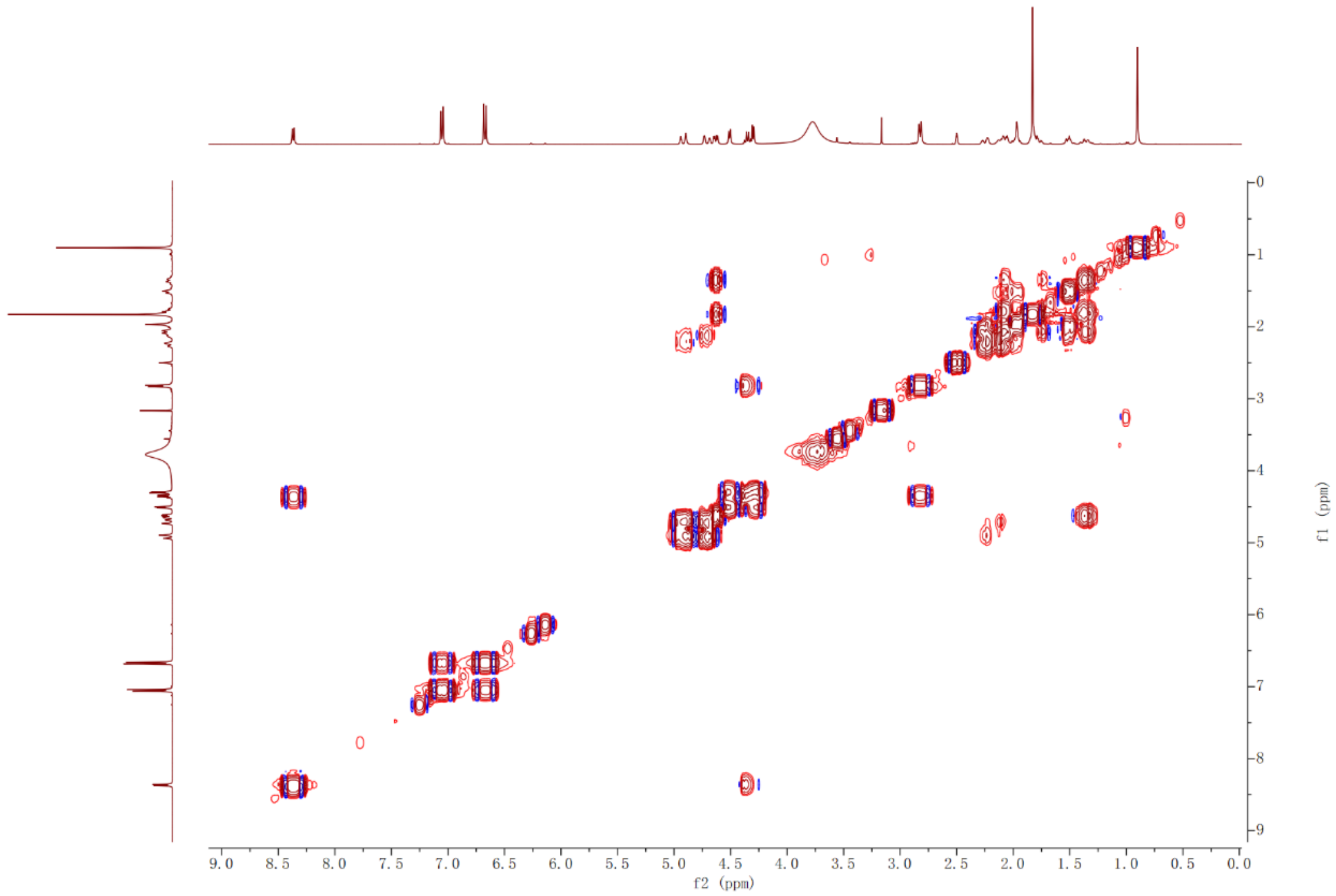

Figure S4. ^{1}H-^{1}H COSY spectrum of **1** in DMSO-d_6

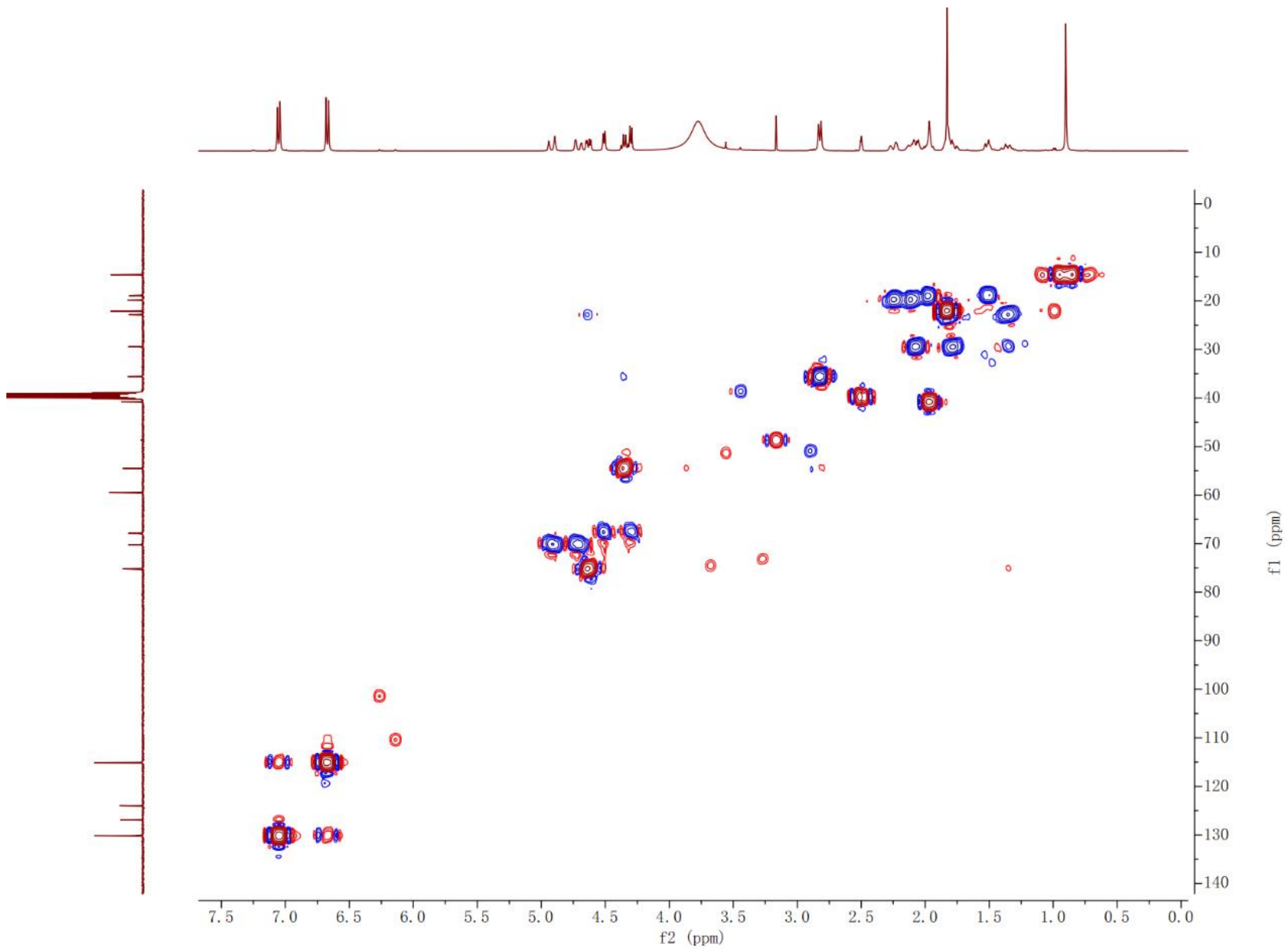

Figure S5. HSQC spectrum of **1** in DMSO-d_6

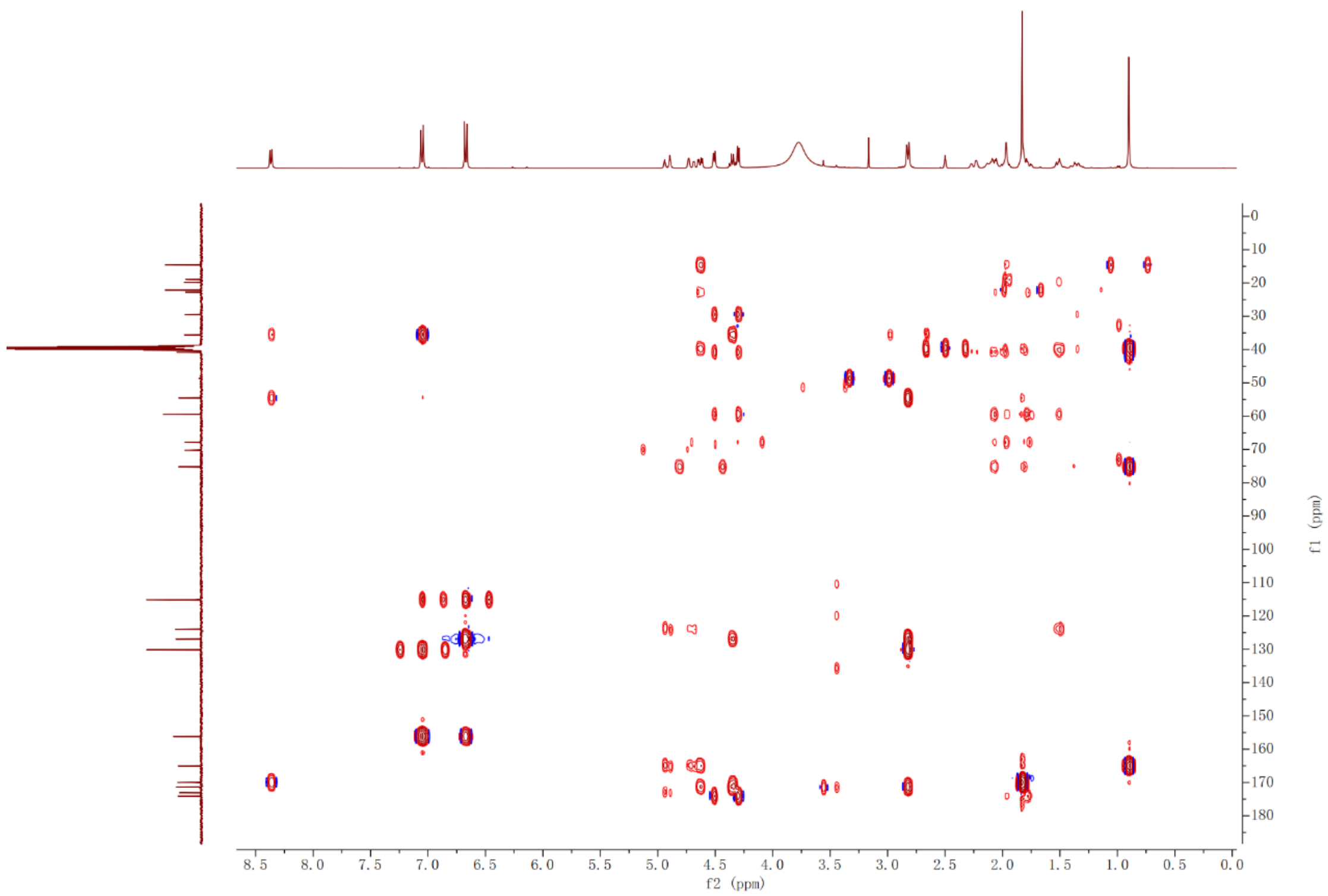

Figure S6. HMBC spectrum of **1** in DMSO-d_6

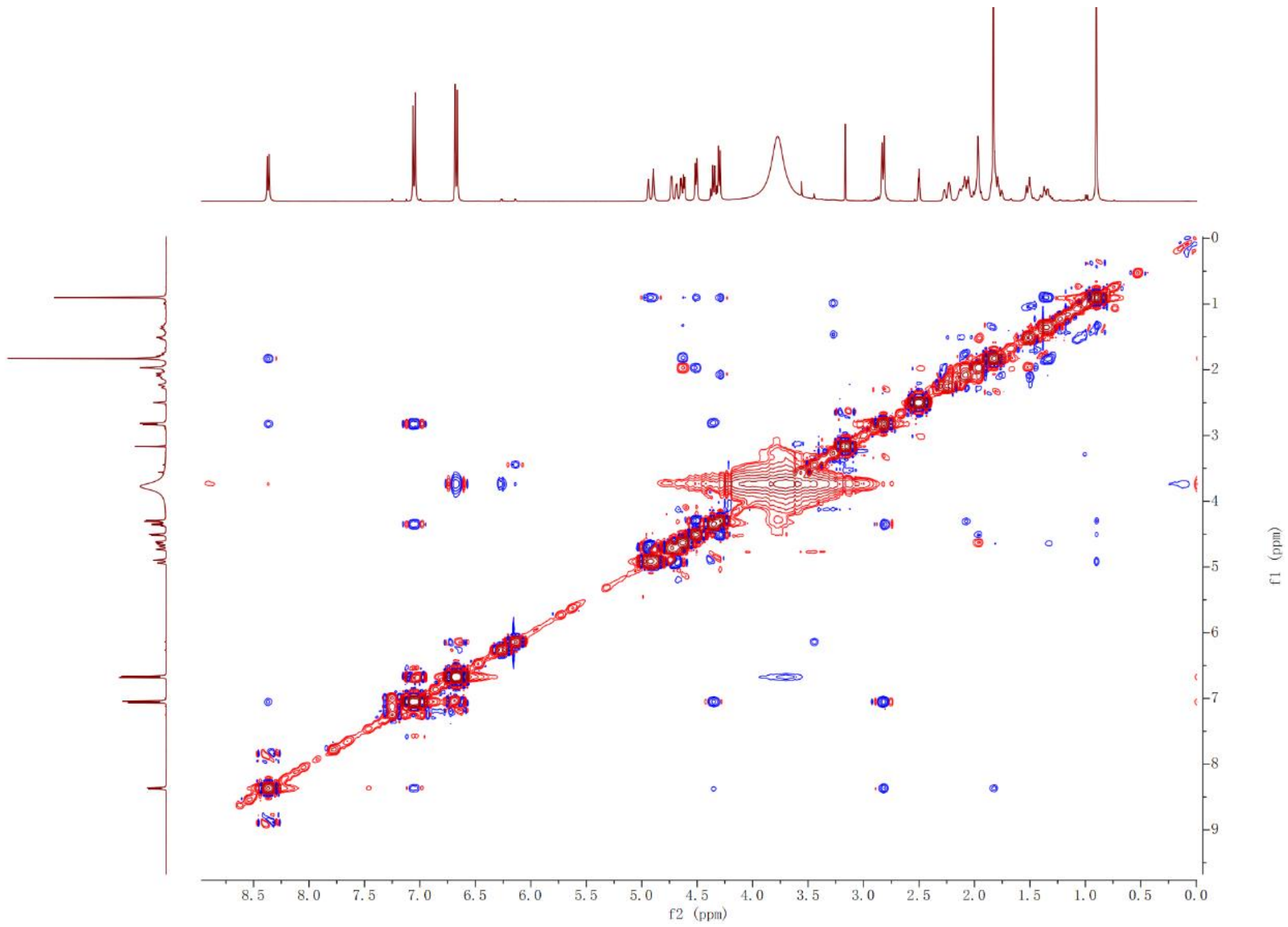

Figure S7. NOESY spectrum of **1** in DMSO-d_6

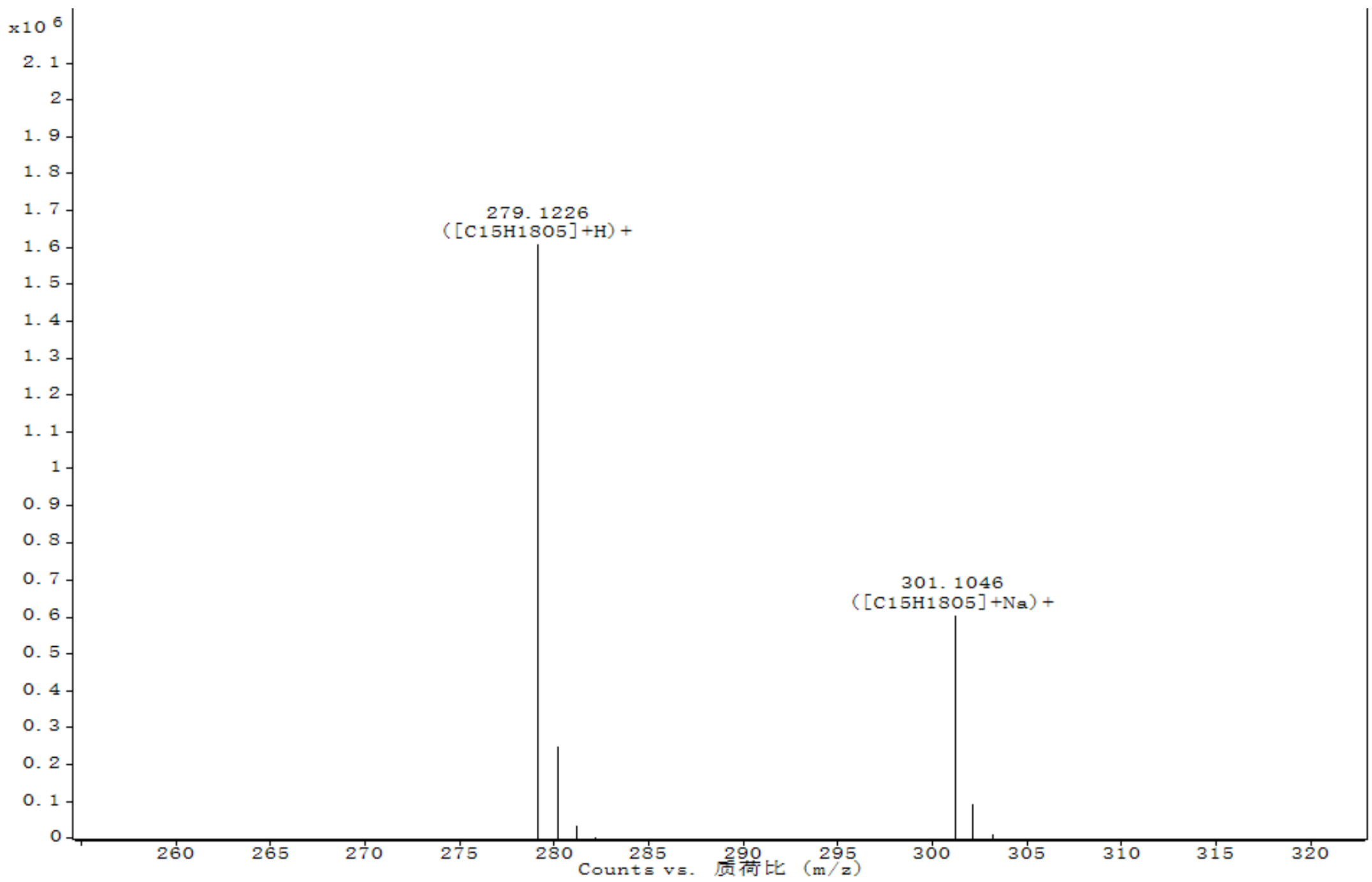

Figure S8. HRESIMS of compound **2**

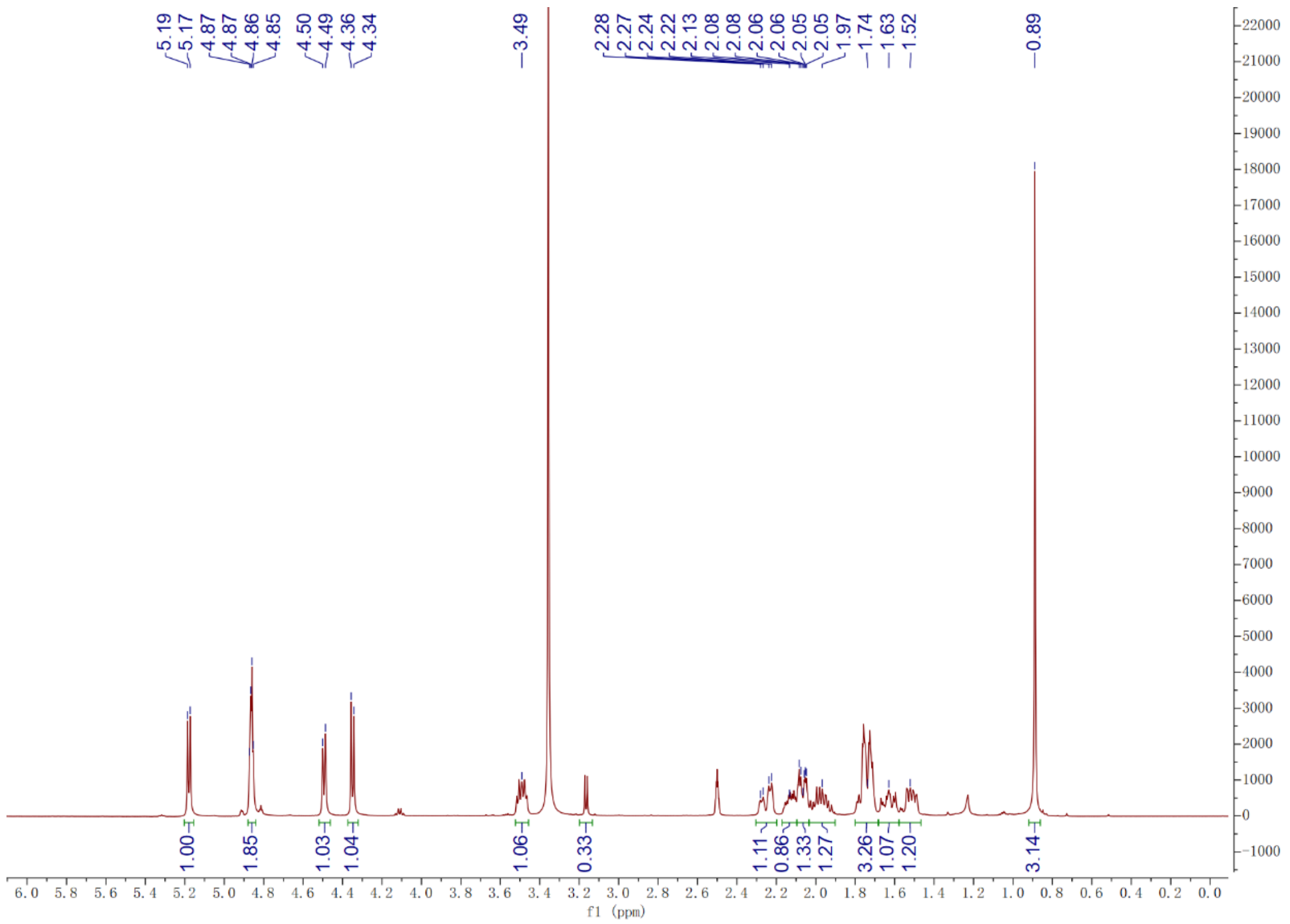

Figure S9. ^{1}H NMR spectrum of **2** in DMSO-d_6

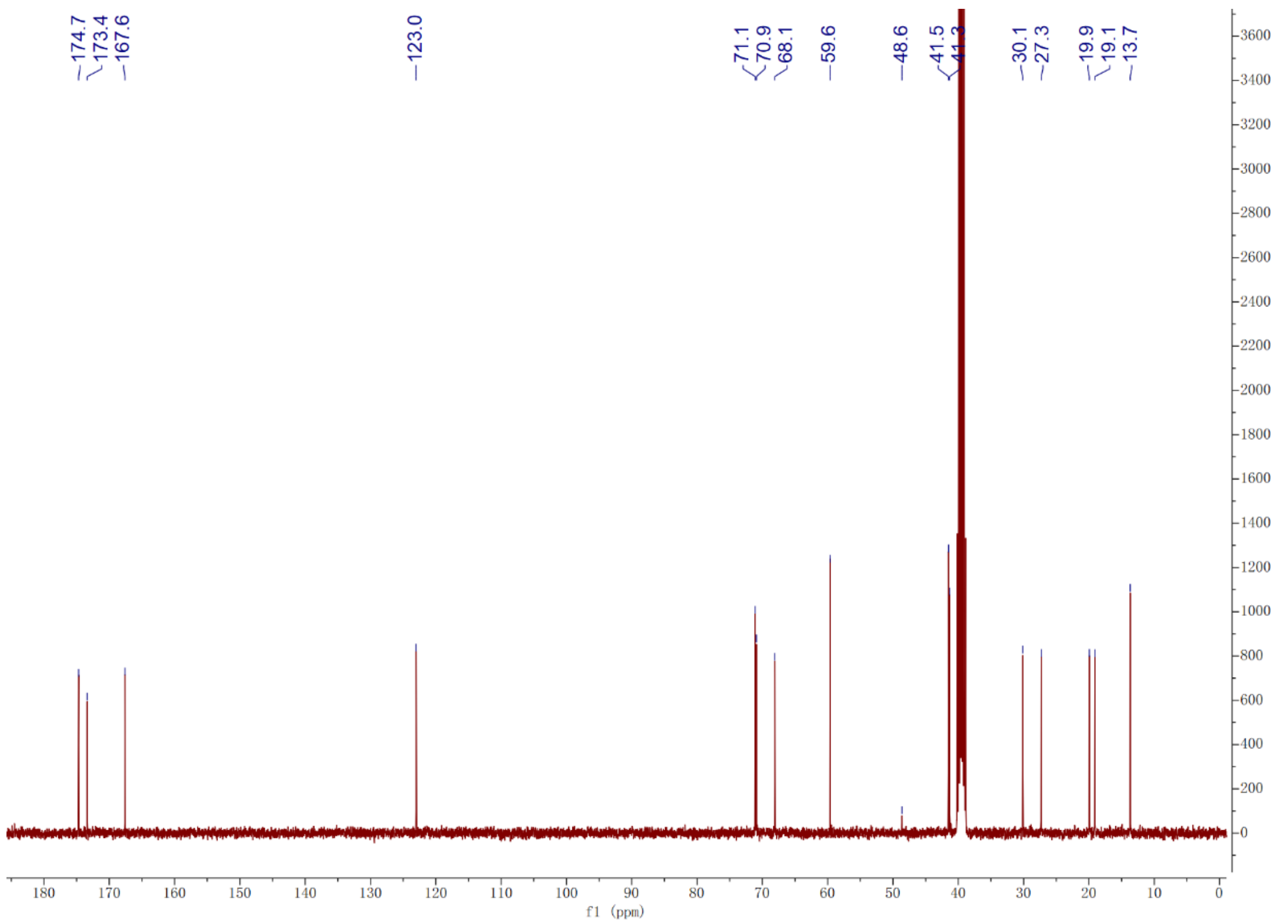

Figure S10. ^{13}C NMR spectrum of **2** in DMSO-d_6

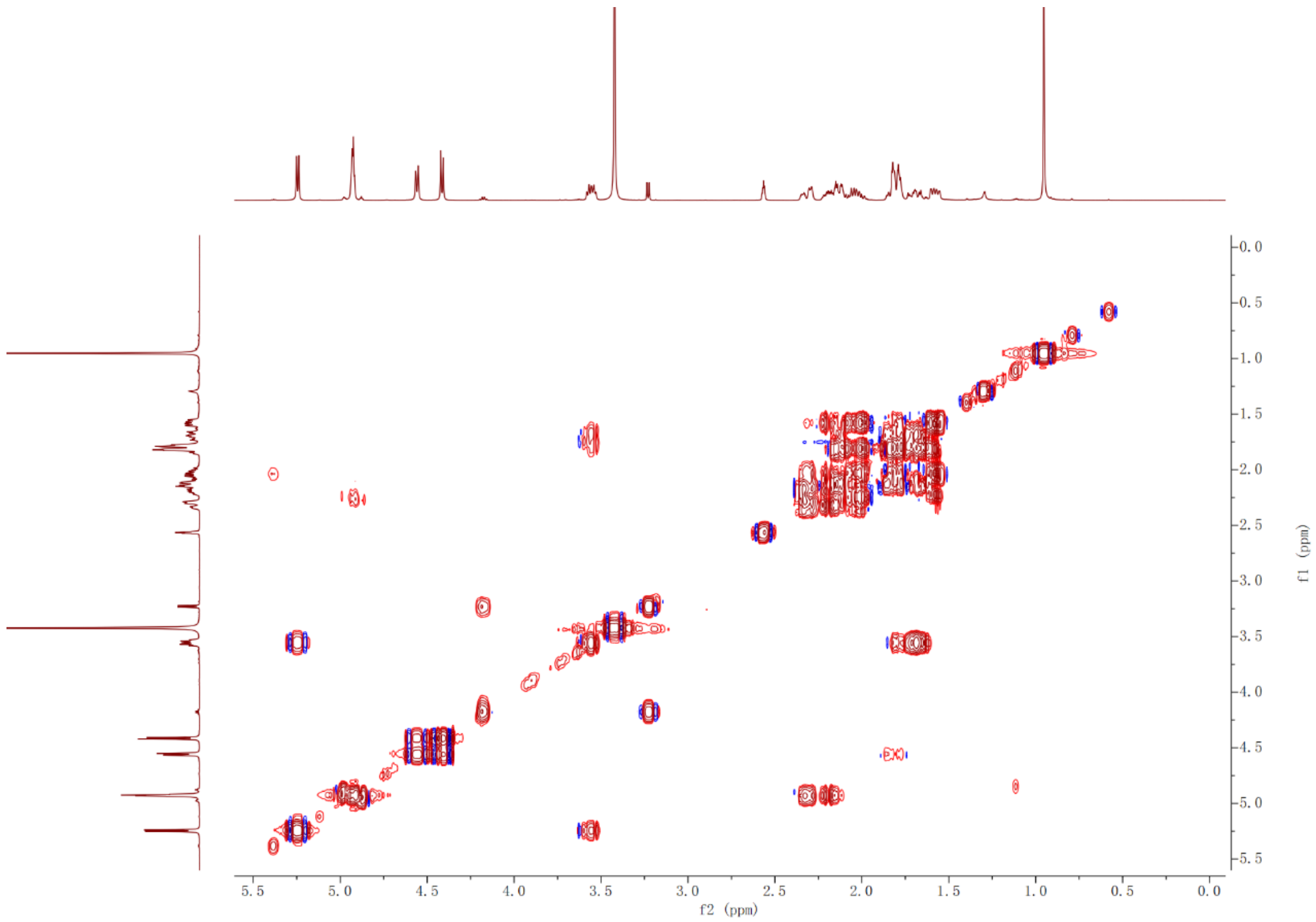

Figure S11. ^{1}H -^{1}H COSY spectrum of **2** in DMSO-d_6

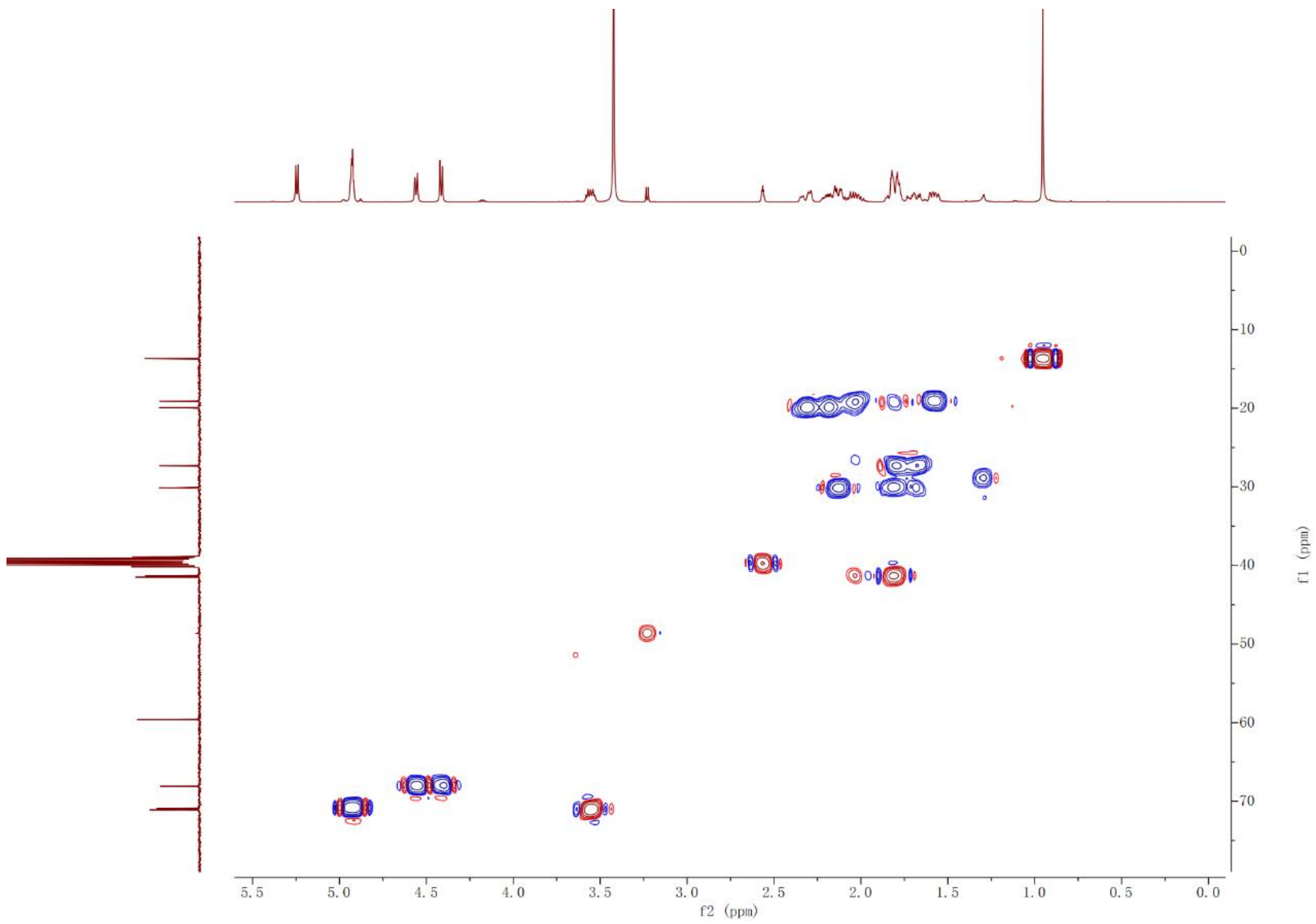

Figure S12. HSQC spectrum of **2** in DMSO-d_6

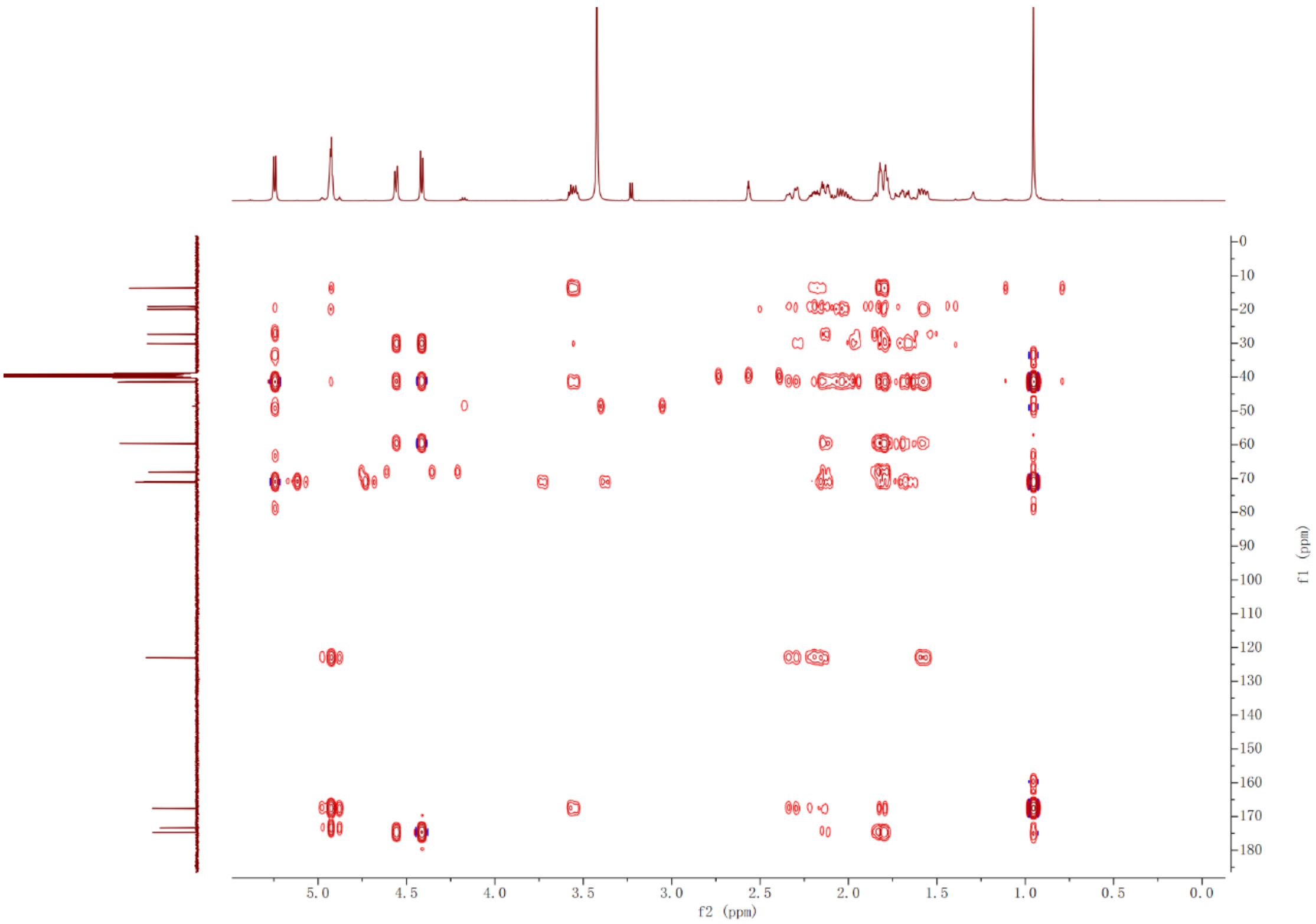

Figure S13. HMBC spectrum of **2** in DMSO-d_6

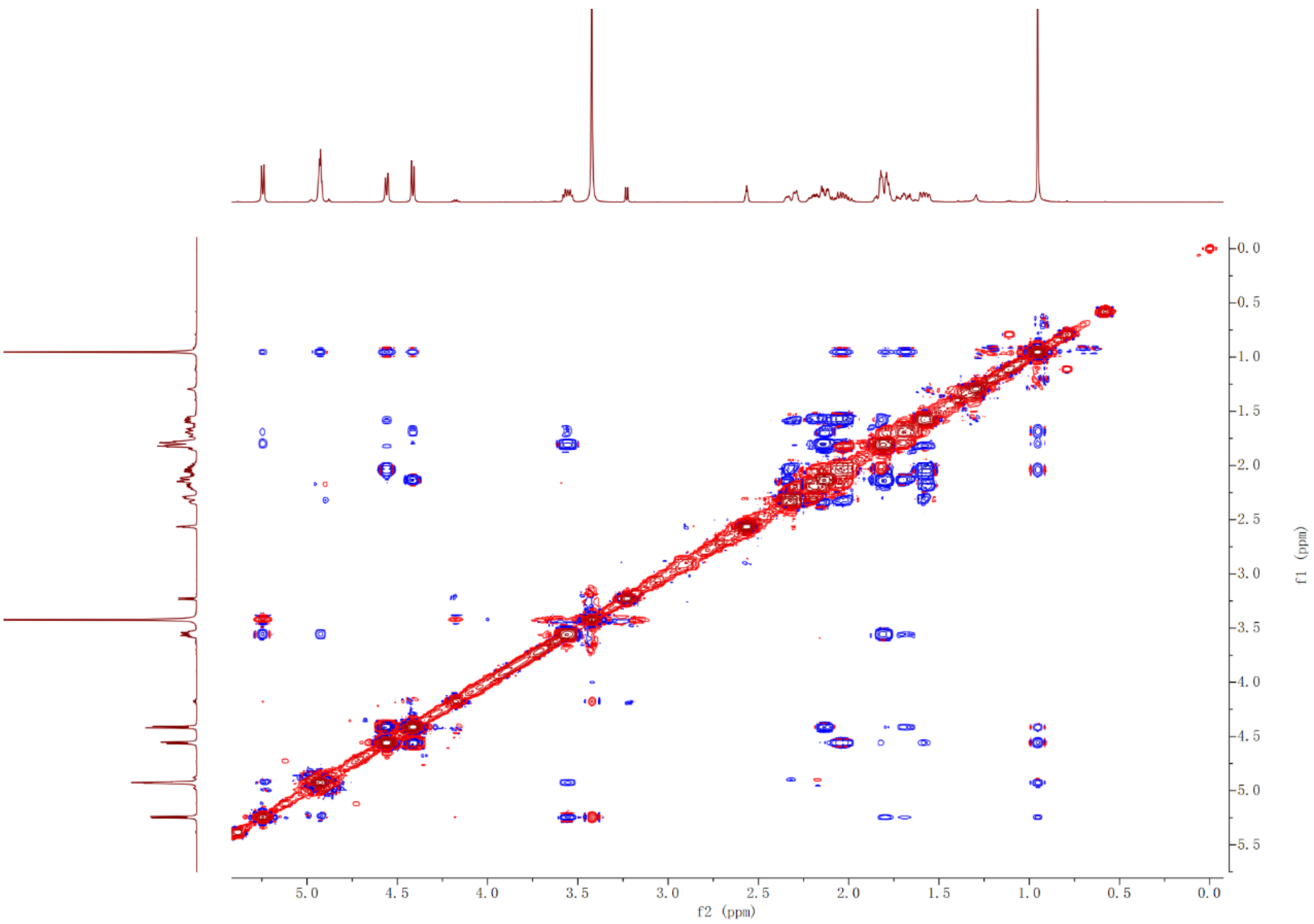

Figure S14. NOESY spectrum of **2** in DMSO-d_6

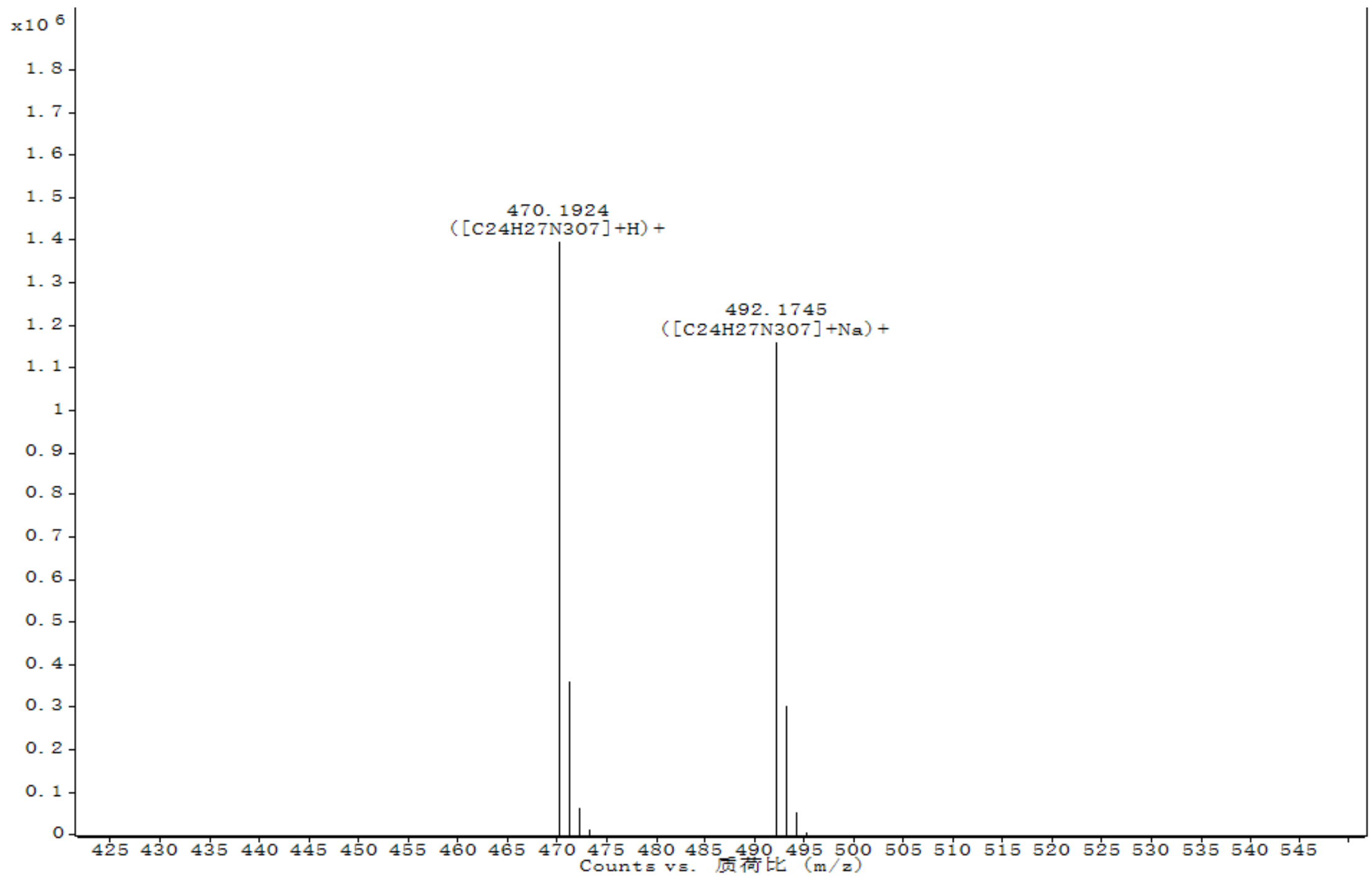

Figure S15. HRESIMS of compound **3**

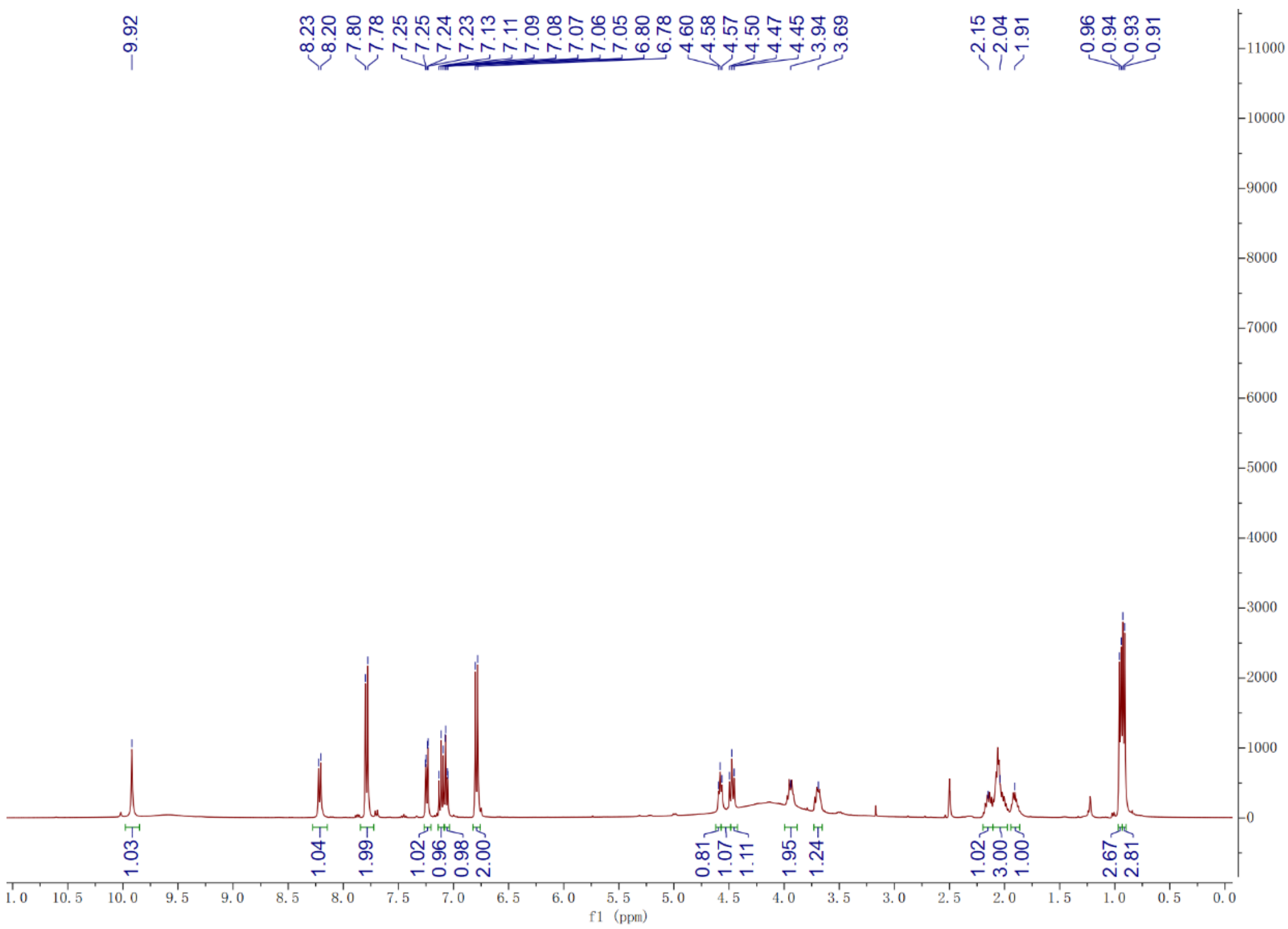

Figure S16. ^{1}H NMR spectrum of **3** in DMSO-d_6

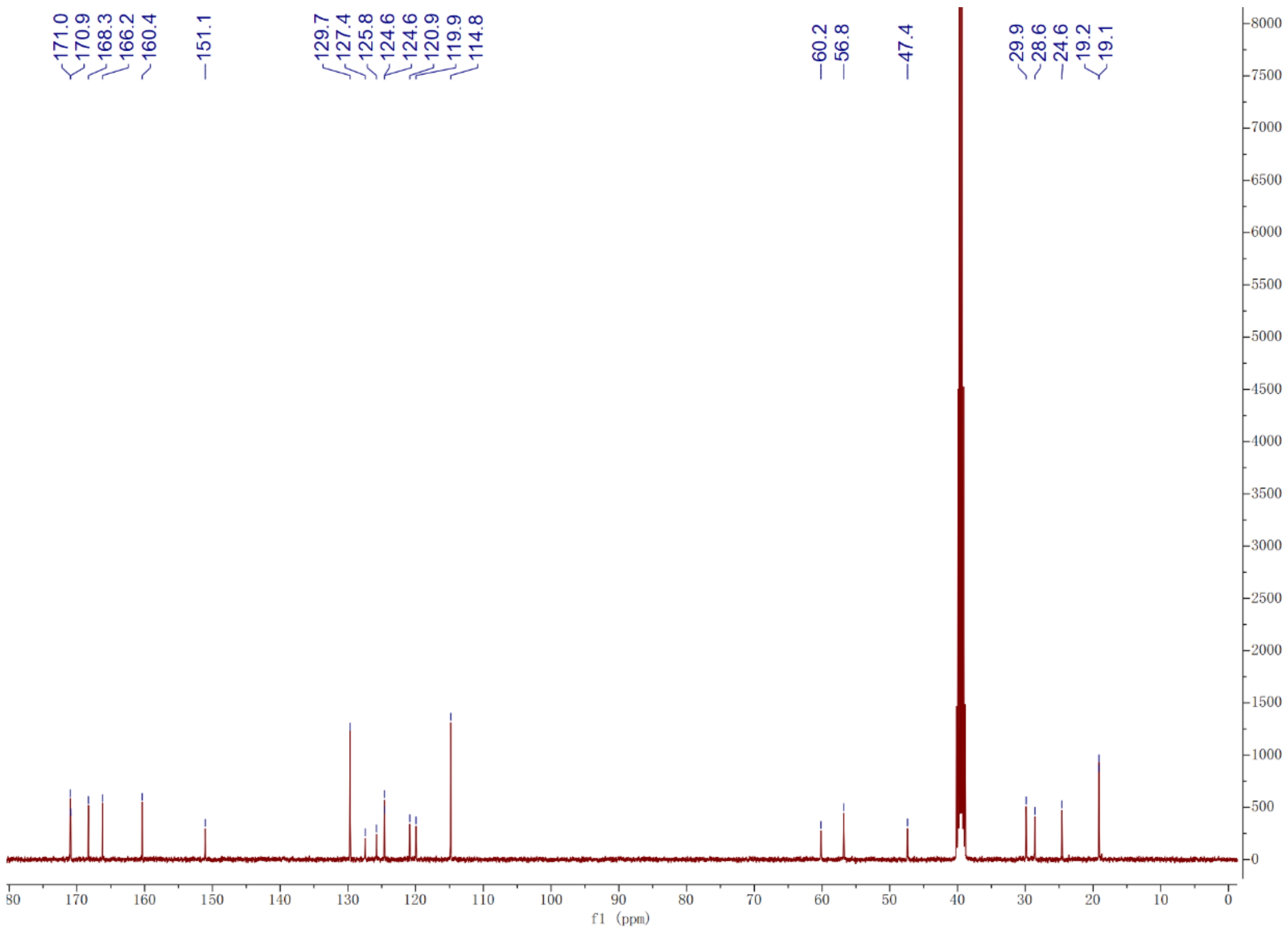

Figure S17. ^{13}C NMR spectrum of **3** in DMSO-d_6

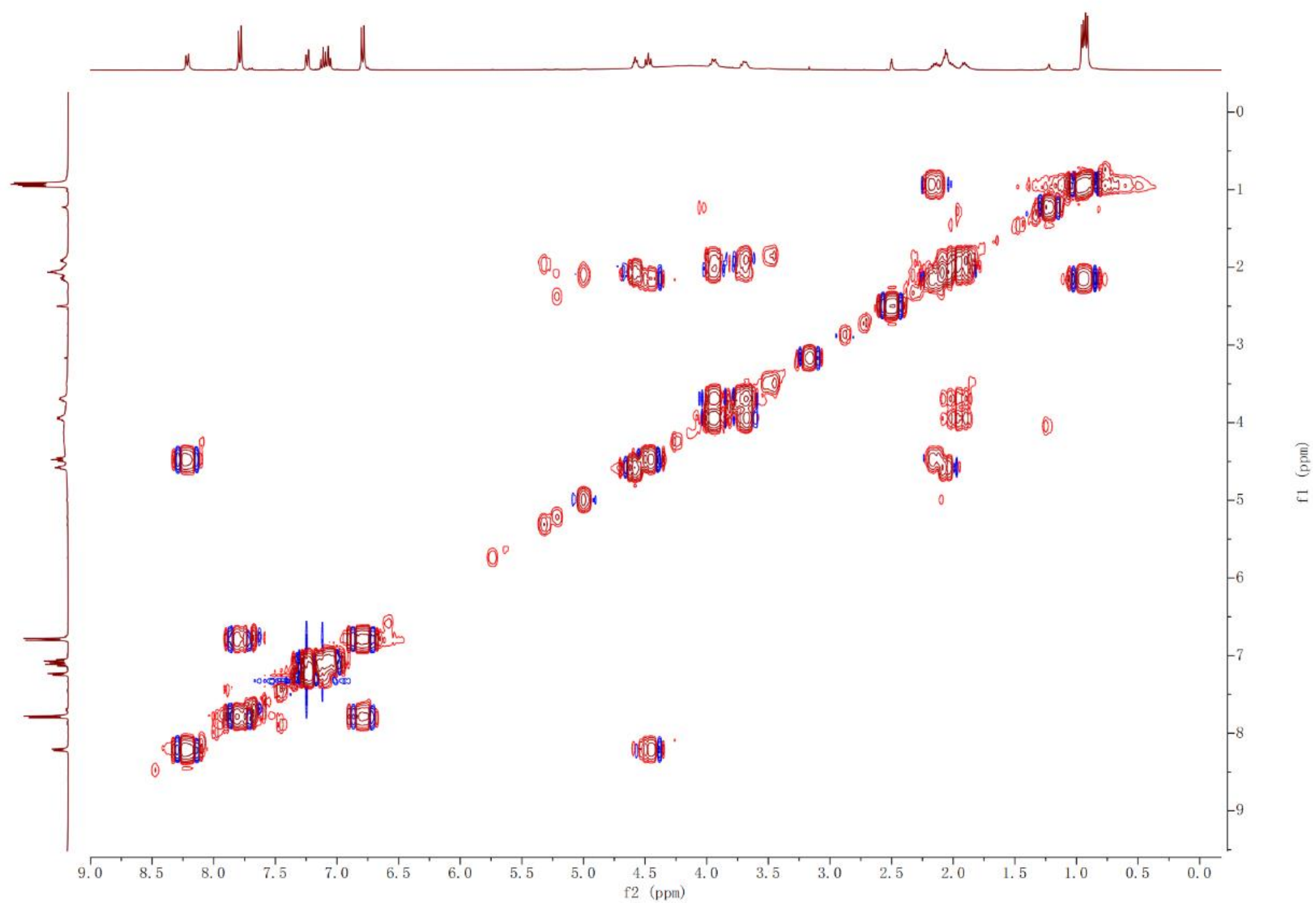

Figure S18. ^1H -^1H COSY spectrum of **3** in DMSO-d_6

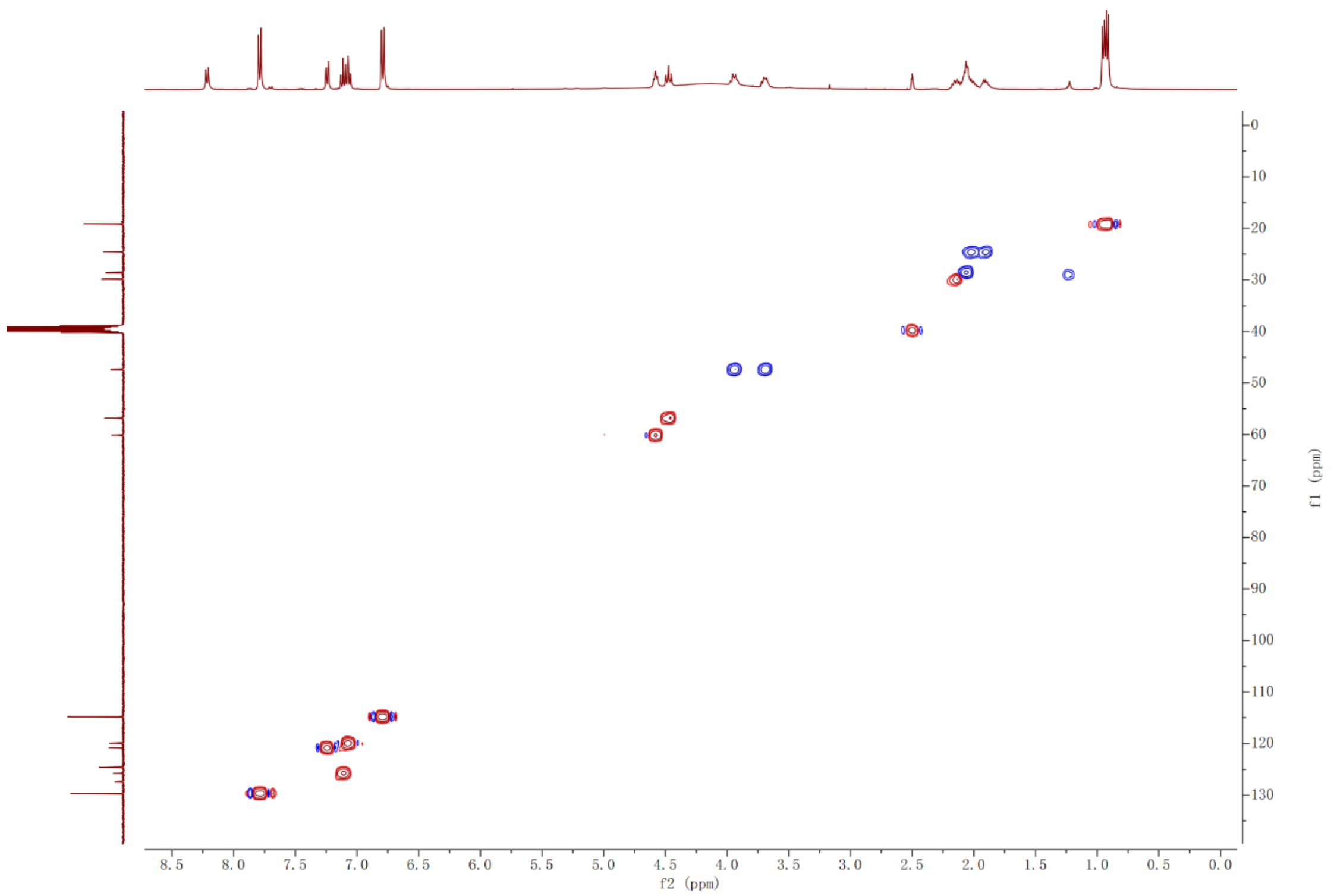

Figure S19. HSQC spectrum of **3** in DMSO-d_6

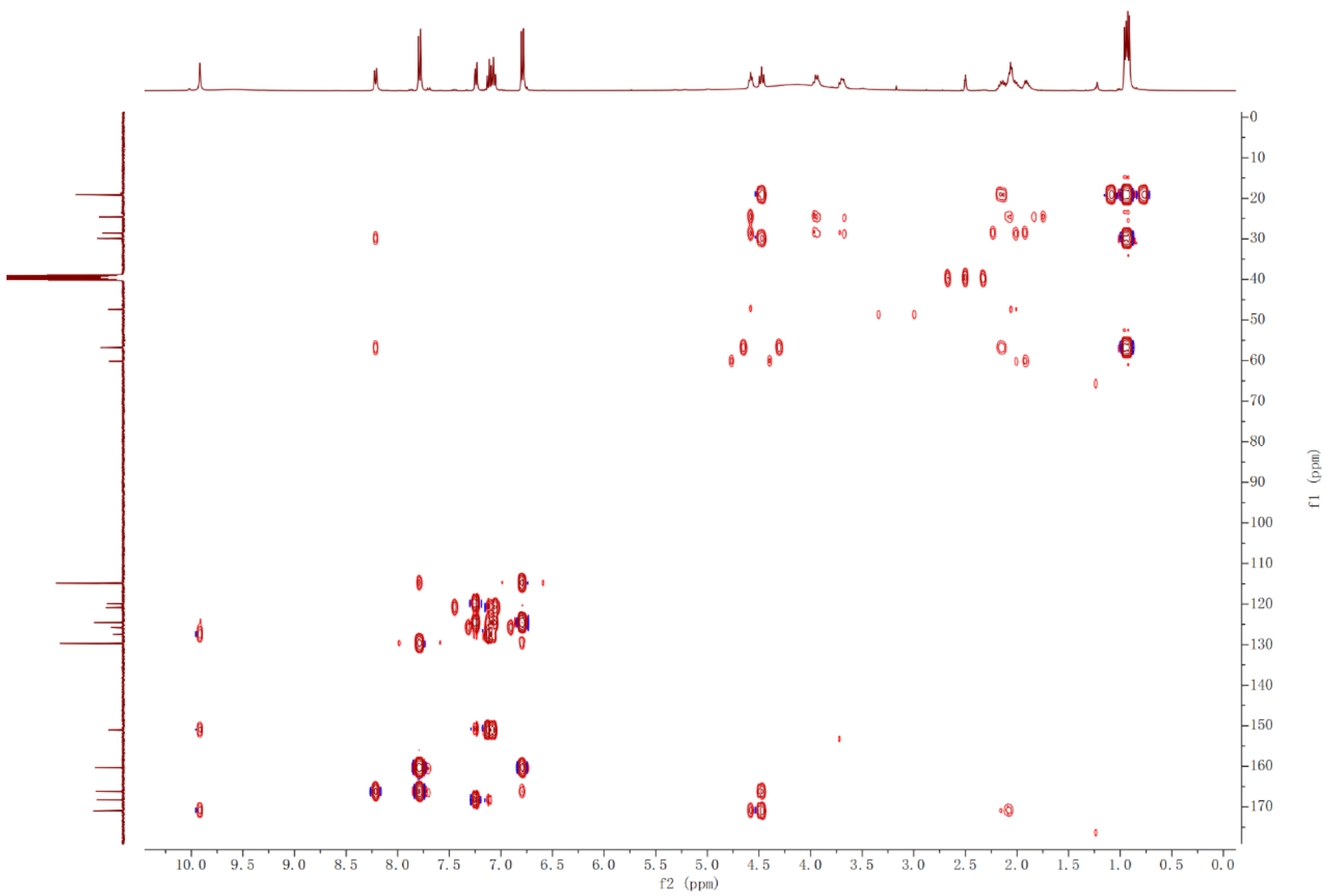

Figure S20. HMBC spectrum of **3** in DMSO-d_6

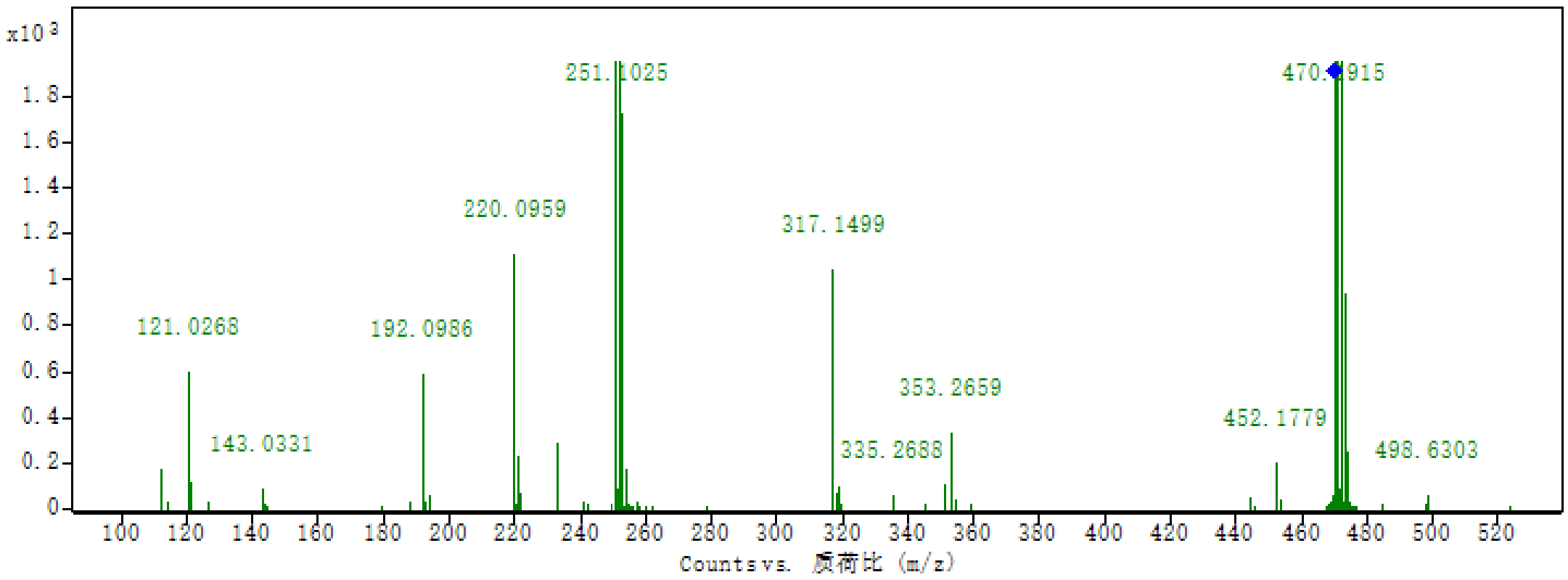

Figure S21. ESI-MS^2 fragmentation of compound **3**

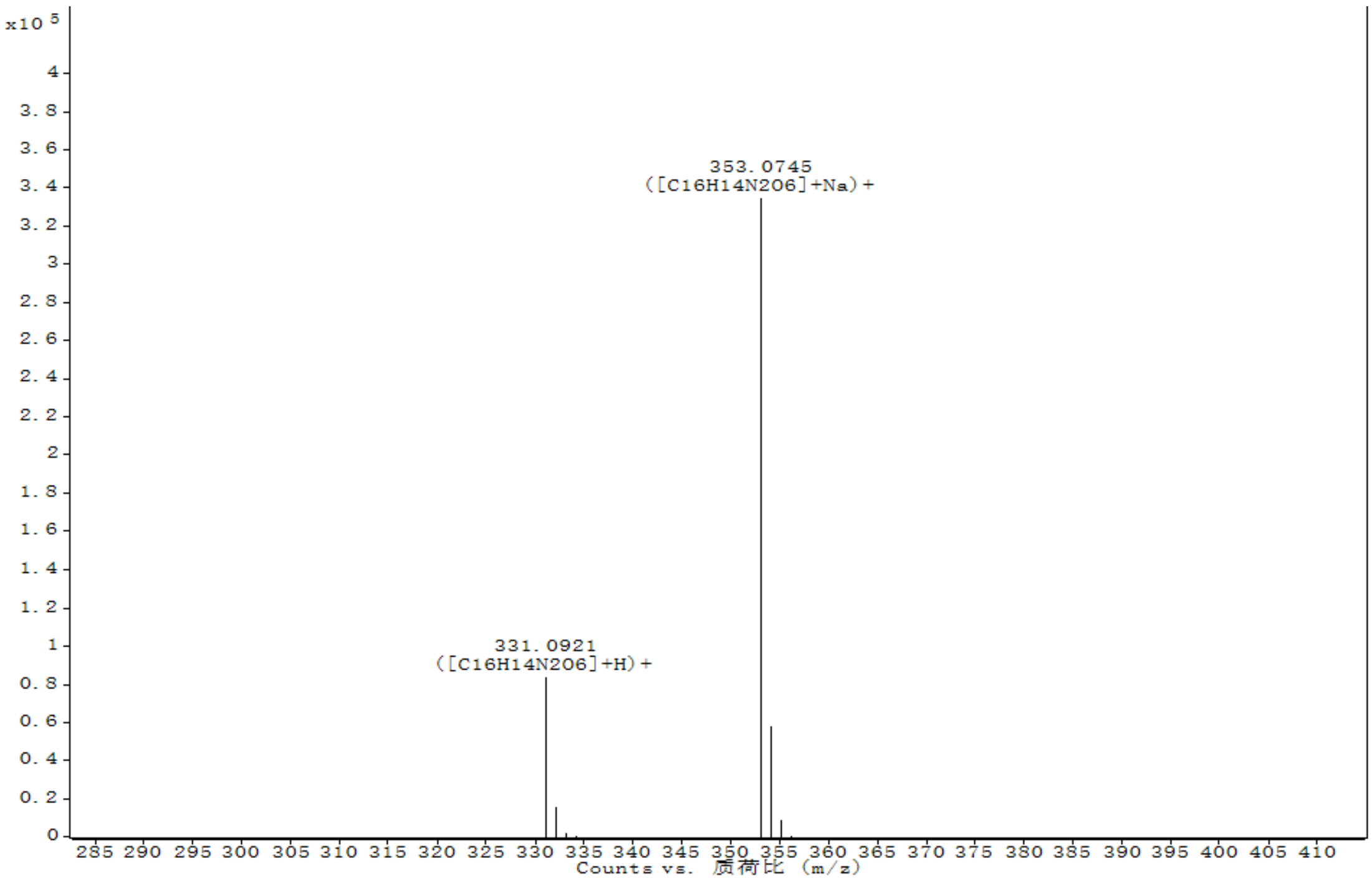

Figure S22. HRESIMS of compound **4**

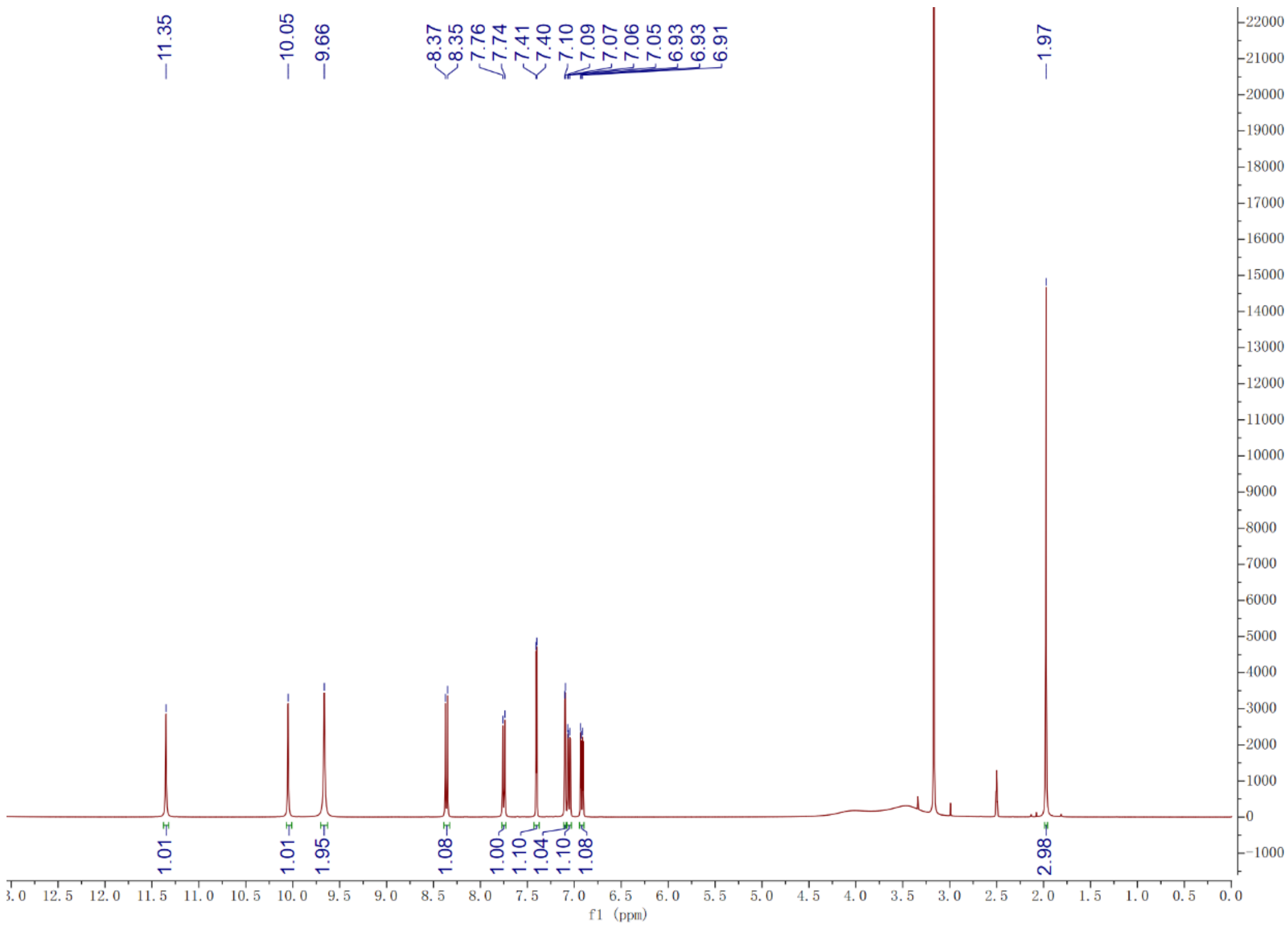

Figure S23. ^{1}H NMR spectrum of **4** in DMSO-d_6

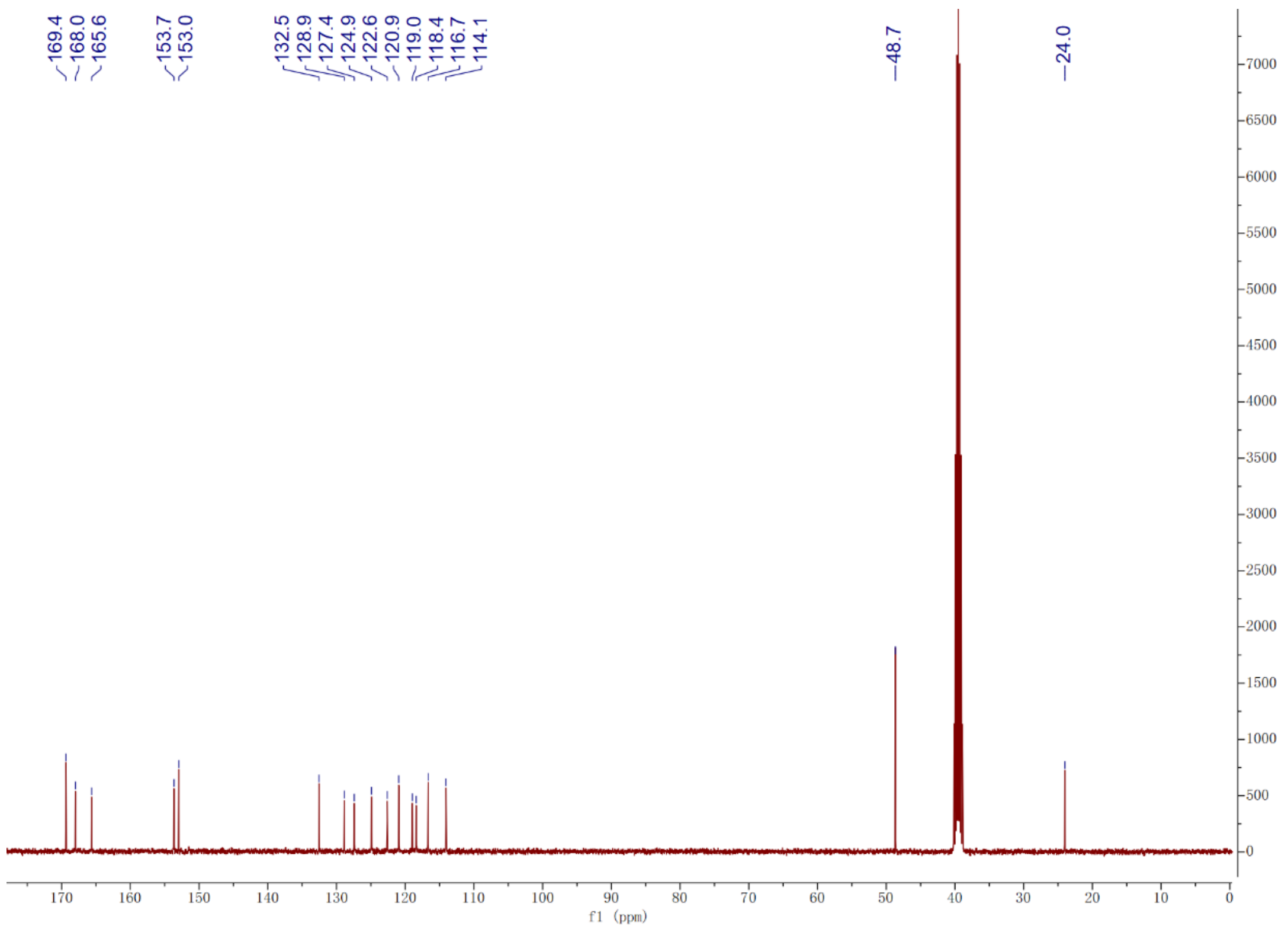

Figure S24. ^{13}C NMR spectrum of **4** in DMSO-d_6

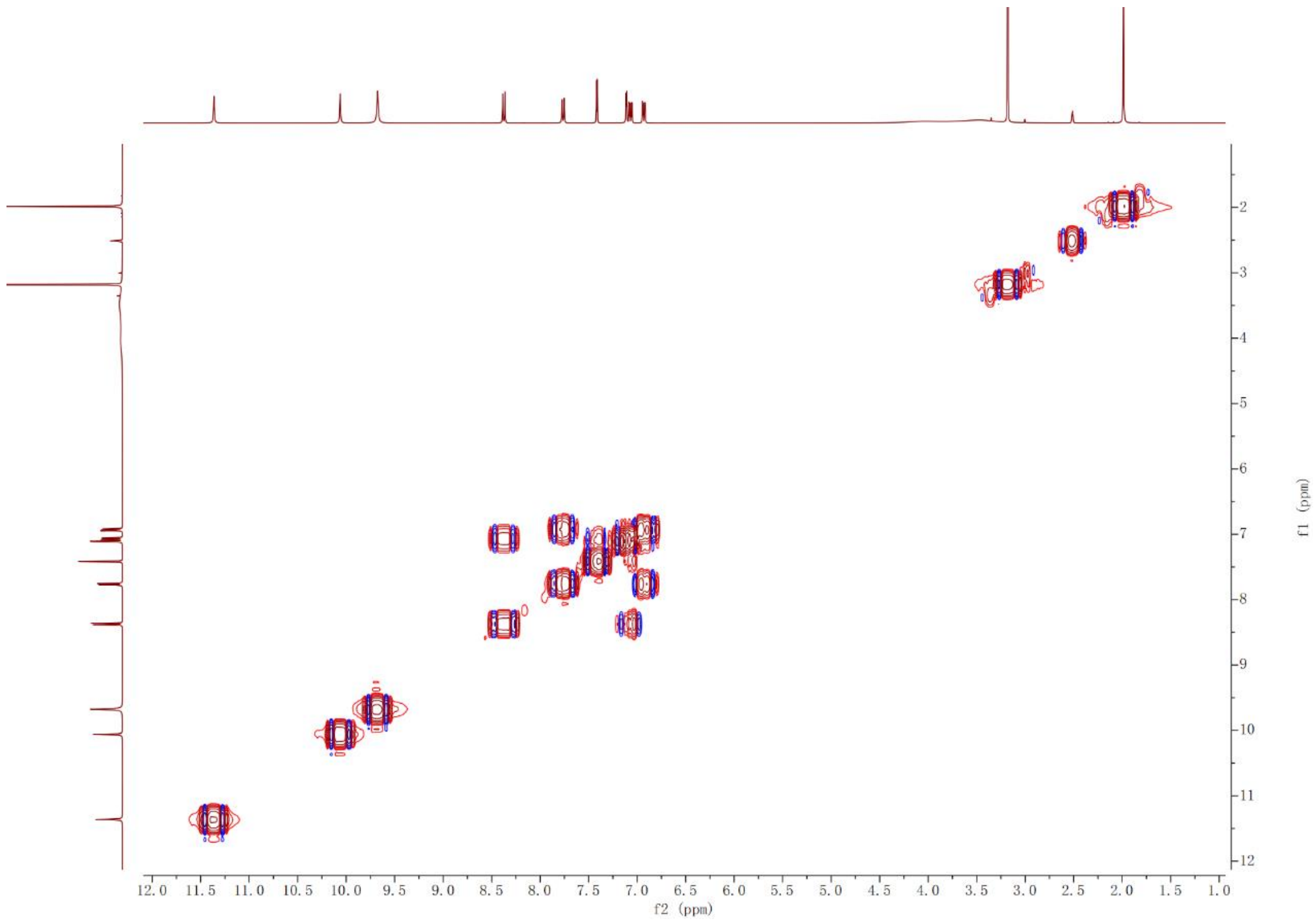

Figure S25. ^{1}H -^{1}H COSY spectrum of **4** in DMSO-d_6

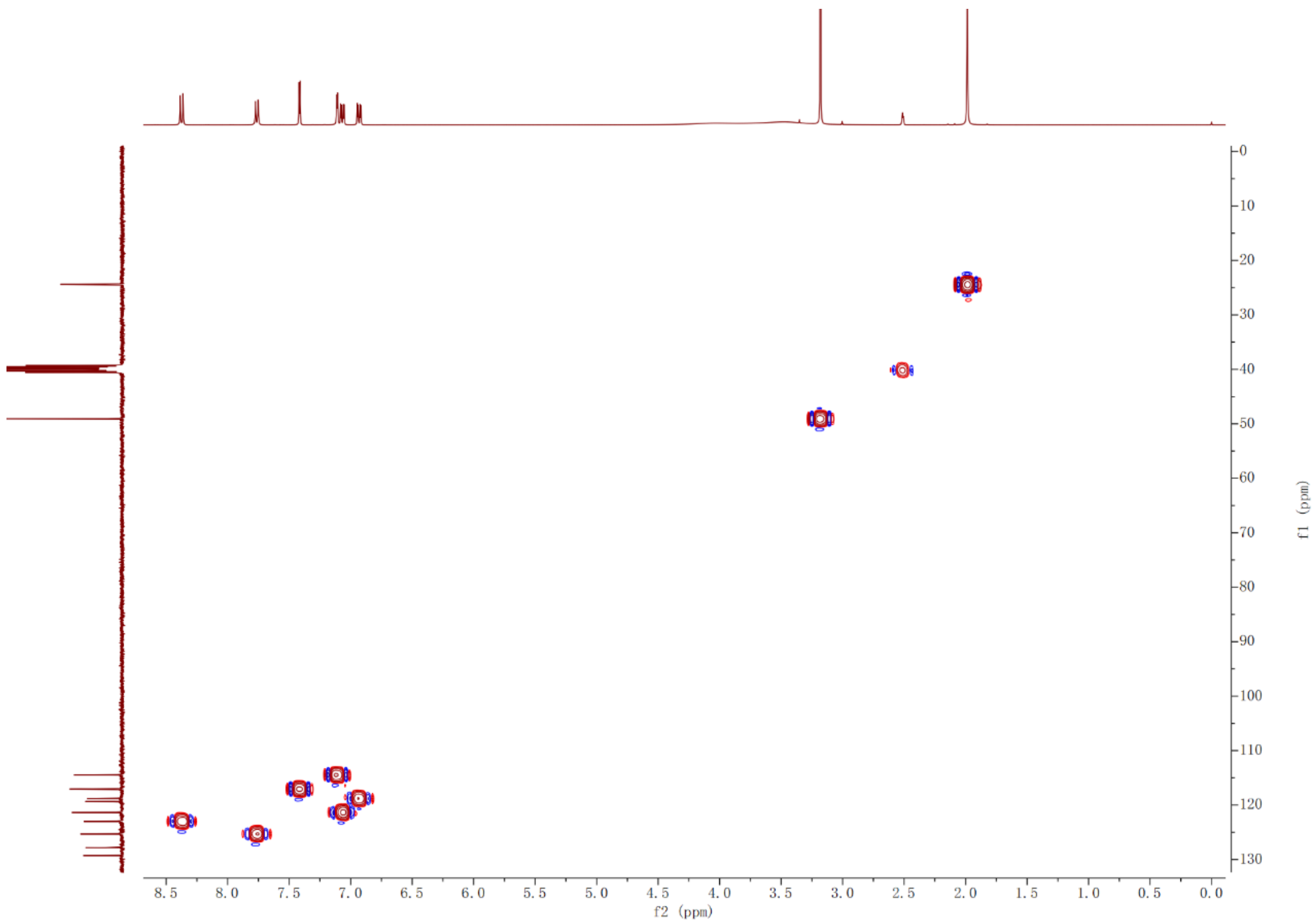

Figure S26. HSQC spectrum of **4** in DMSO-d_6

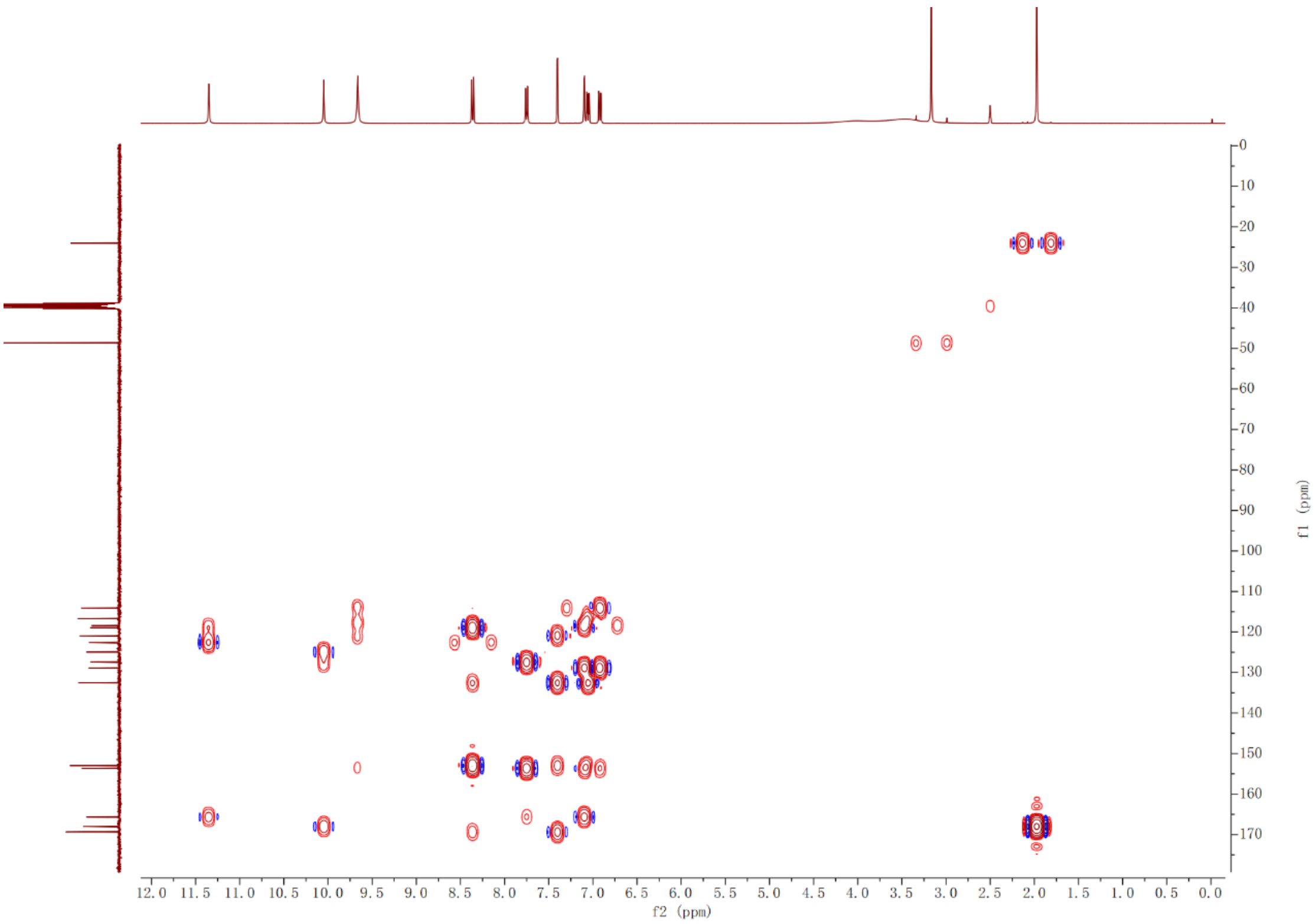

Figure S27. HMBC spectrum of **4** in DMSO-d_6

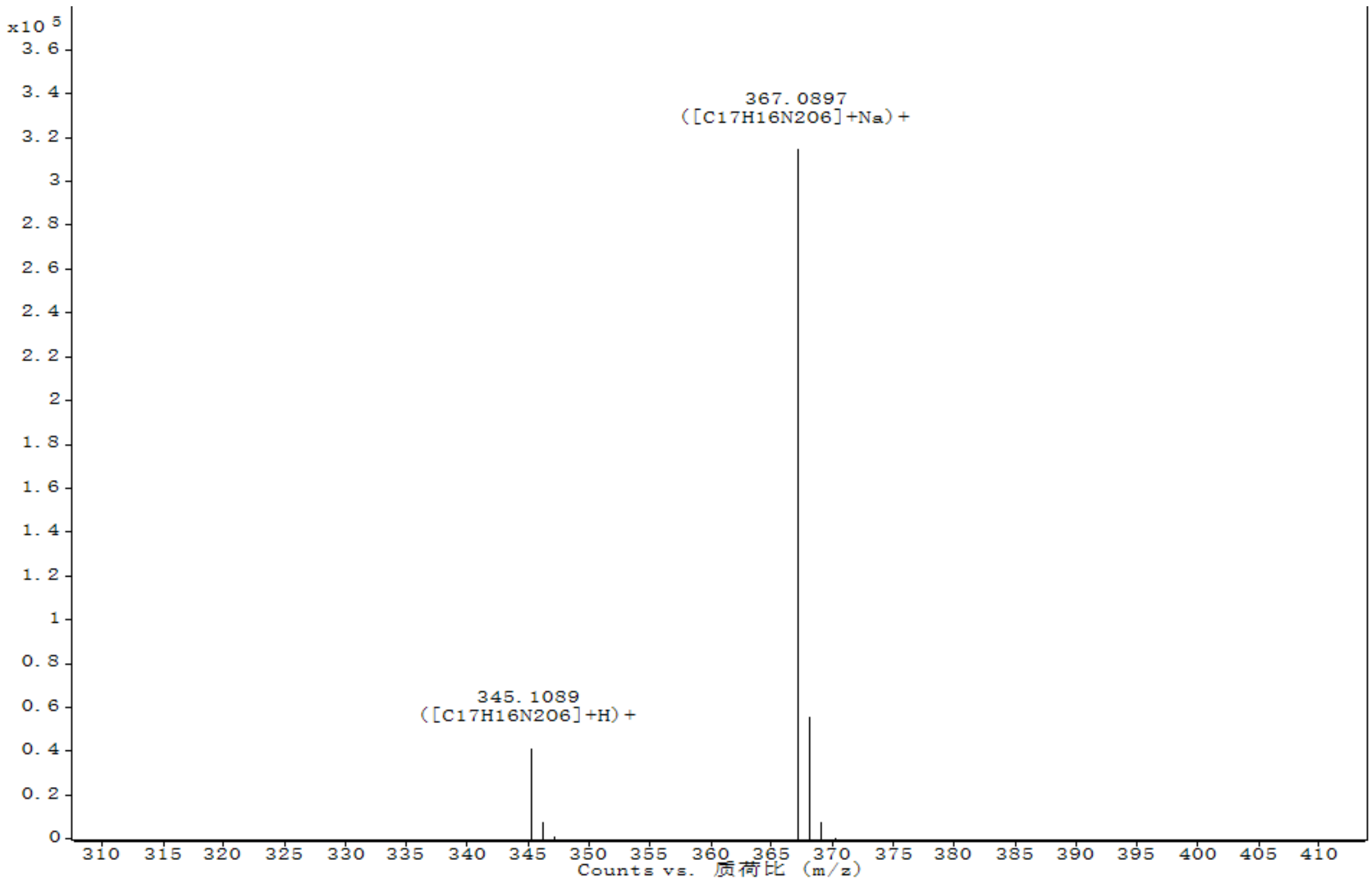

Figure S28. HRESIMS of compound **5**

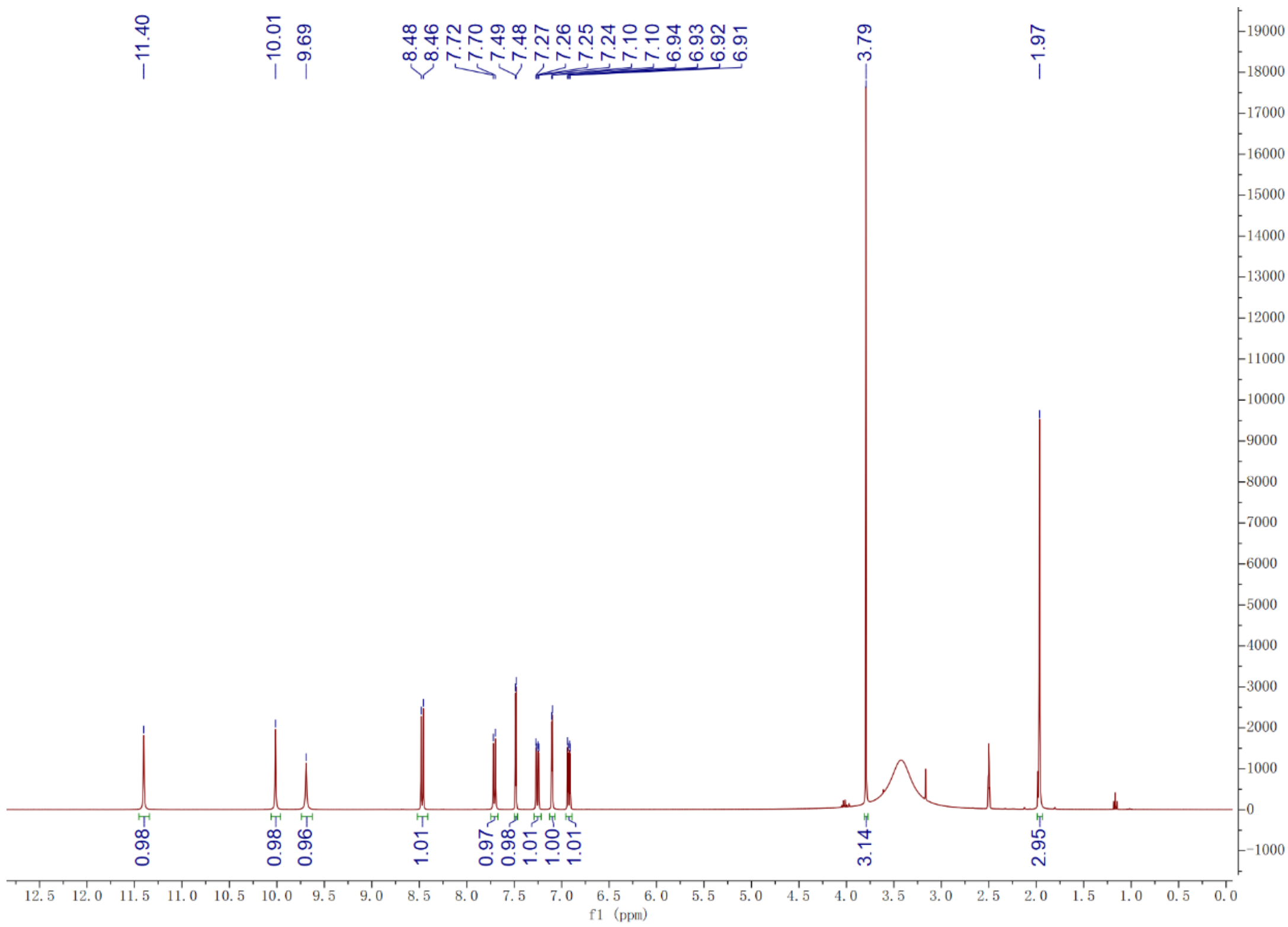

Figure S29. ^{1}H NMR spectrum of **5** in DMSO-d_6

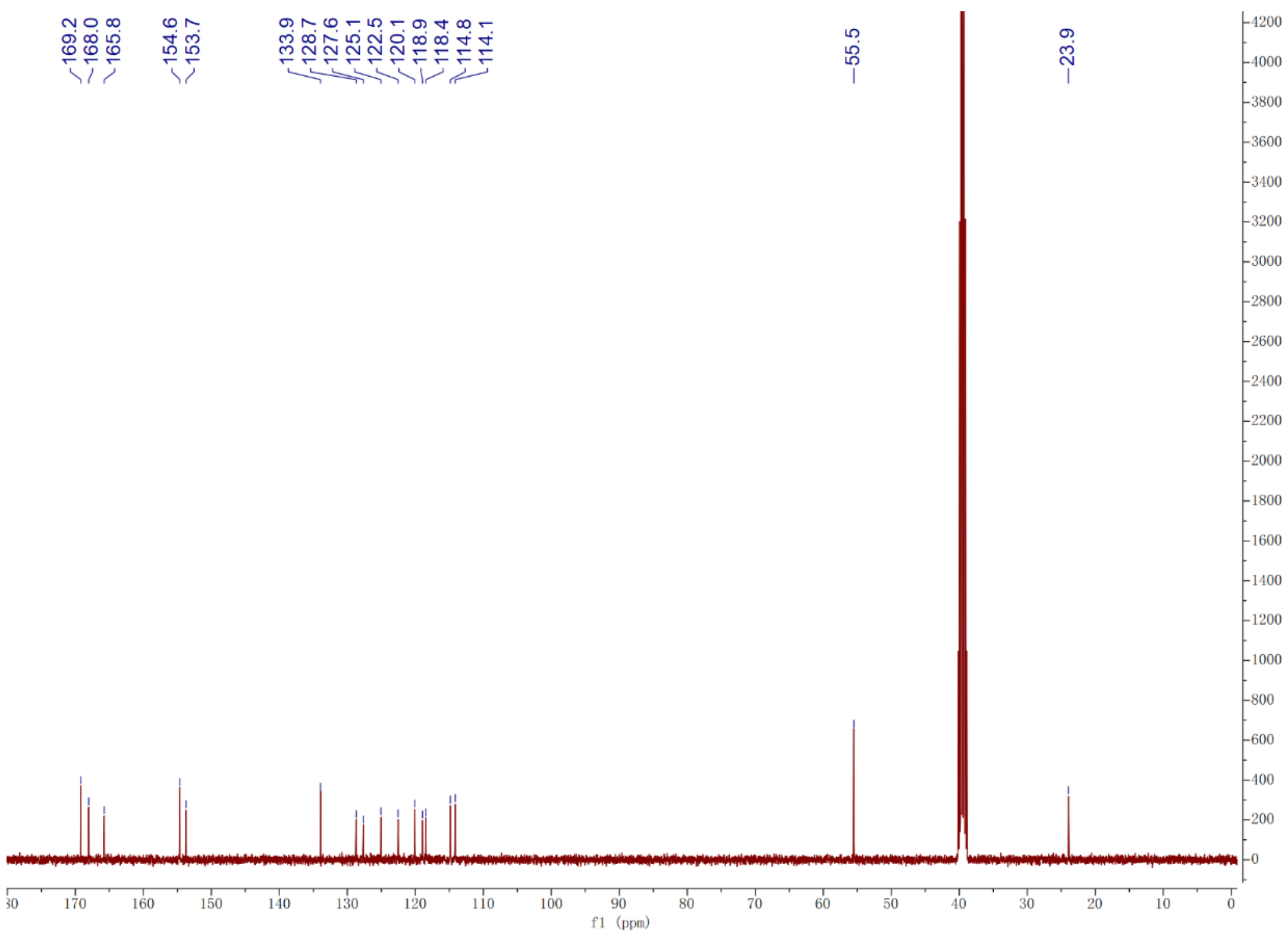

Figure S30. ^{13}C NMR spectrum of **5** in DMSO-d_6

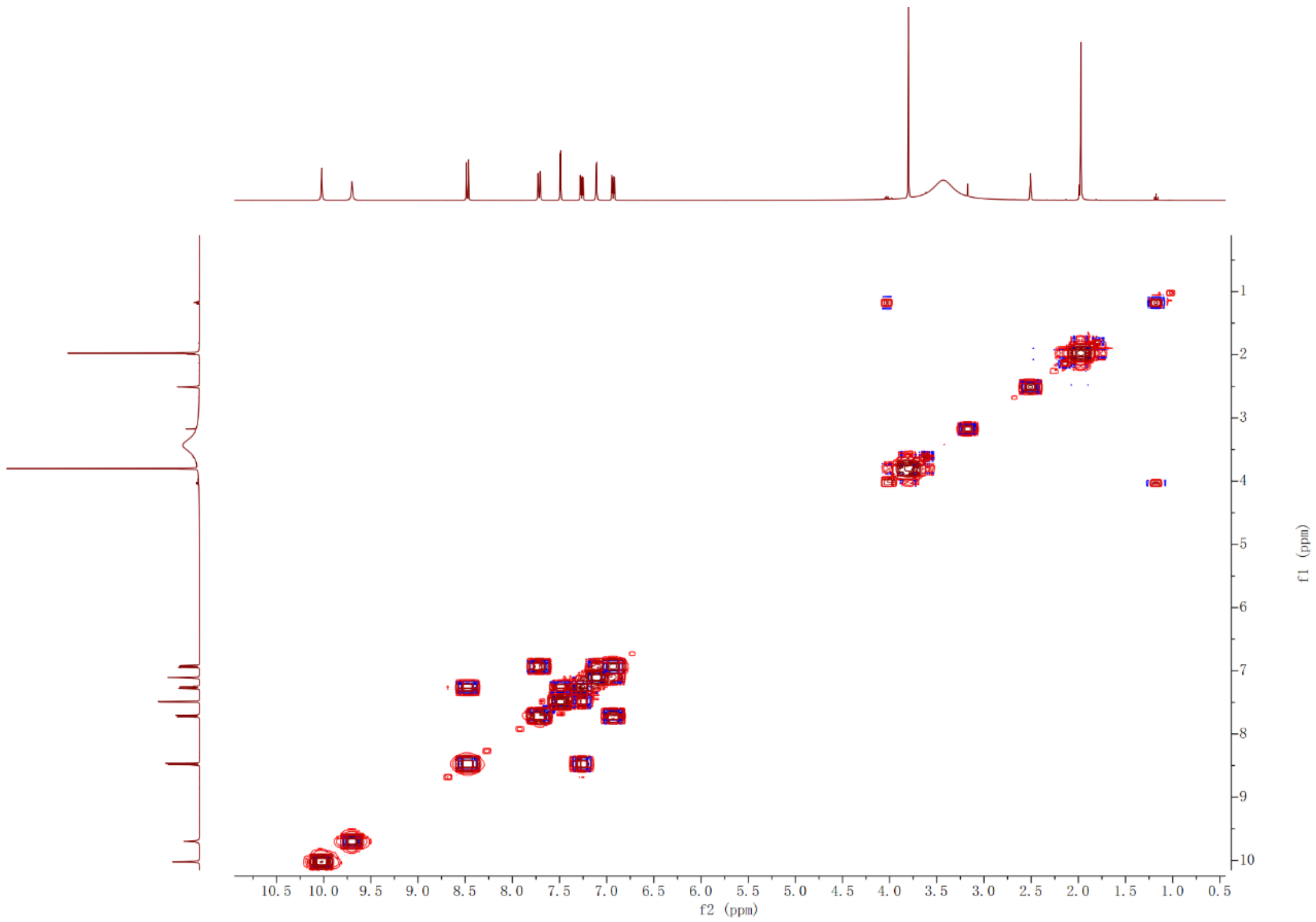

Figure S31. ^{1}H -^{1}H COSY spectrum of **5** in DMSO-d_6

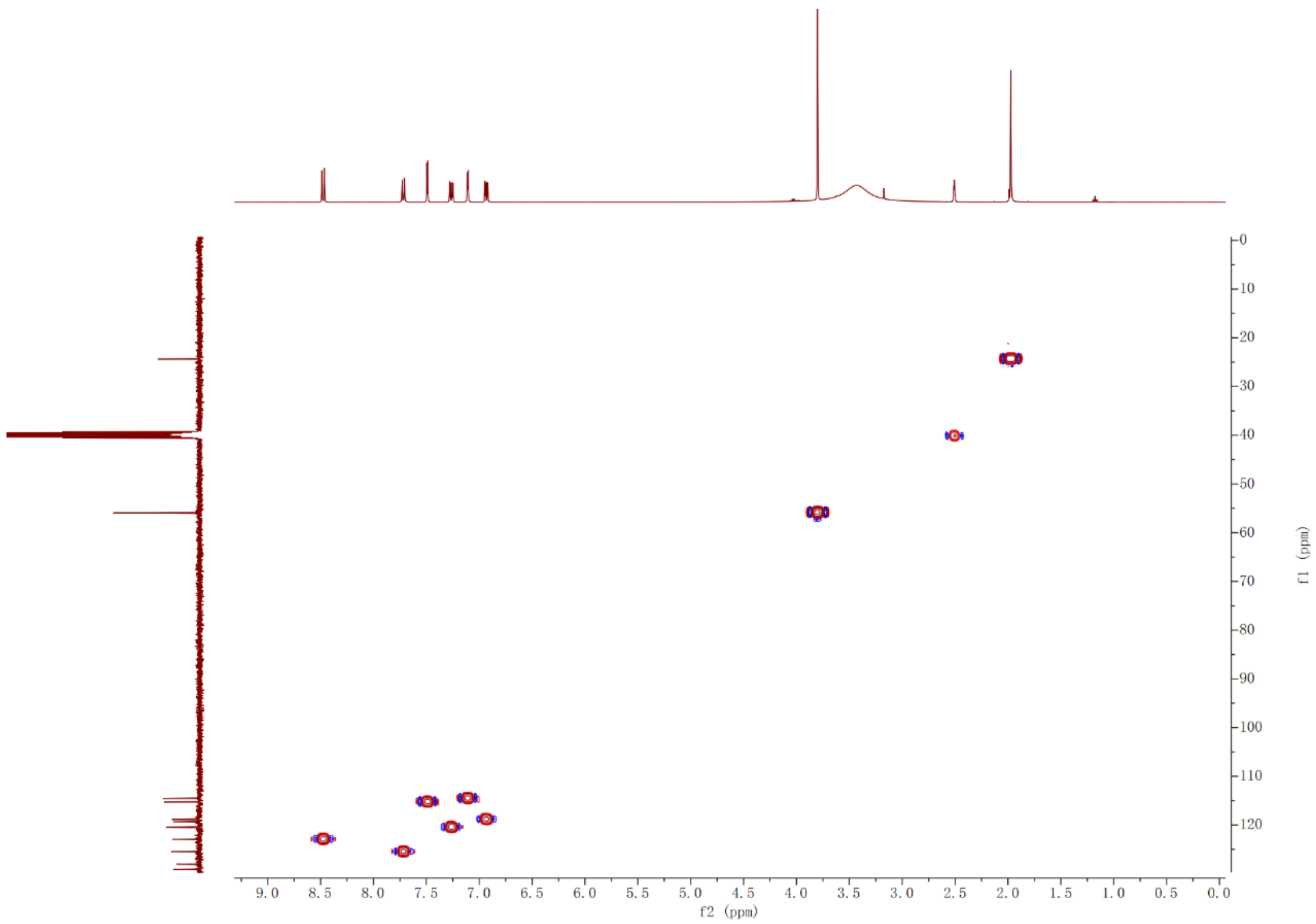

Figure S32. HSQC spectrum of **5** in DMSO-d_6

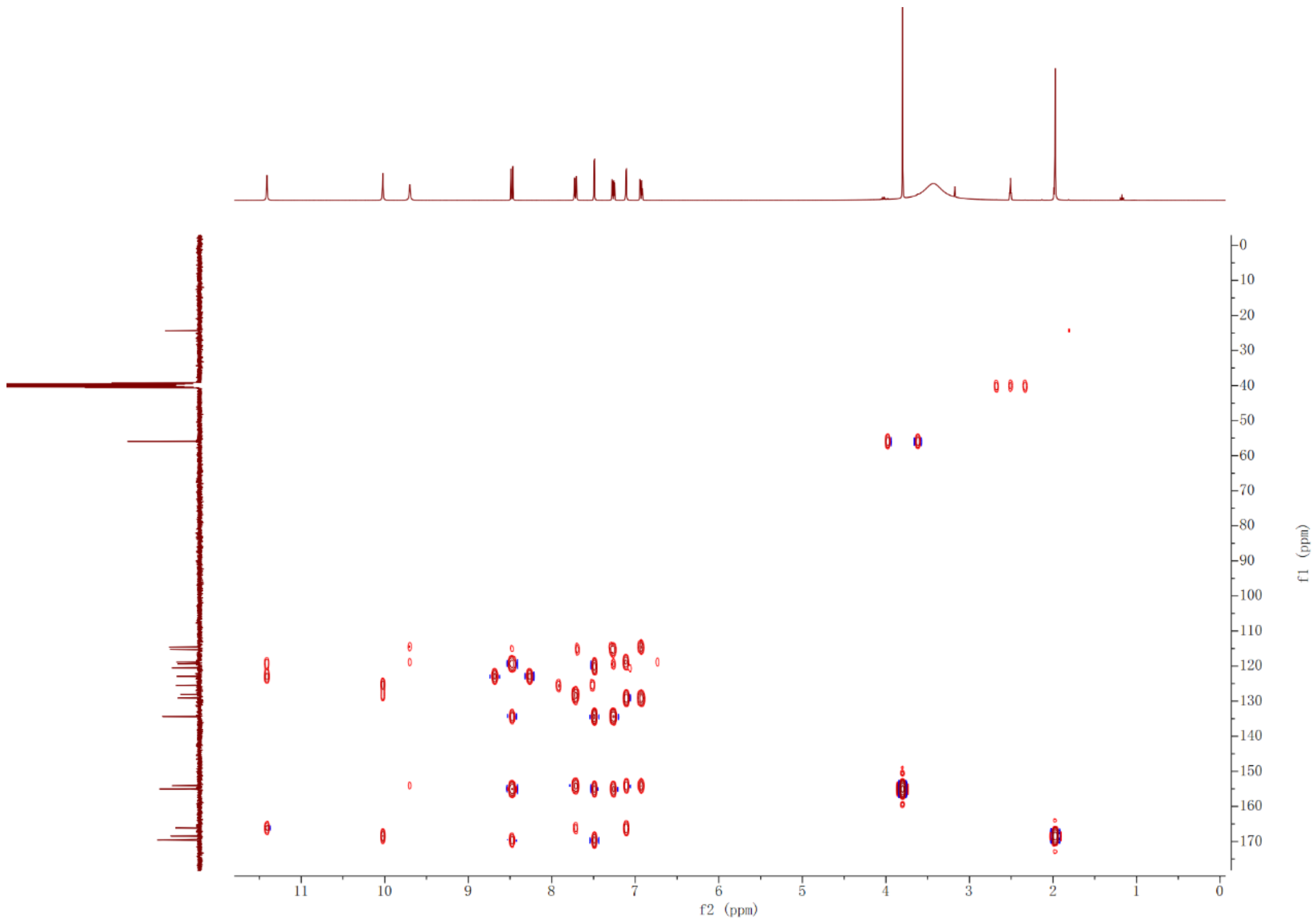

Figure S33. HMBC spectrum of **5** in DMSO-d_6

Table S1. Crystal data and structure refinement for **1**

Identification code	**1**
Empirical formula	$C_{28}H_{37}NO_{10}$
Formula weight	547.58
Temperature/K	277(2)
Crystal system	orthorhombic
Space group	$P2_12_12_1$
a/Å	10.5892(10)
b/Å	11.81890(10)
c/Å	22.2075(3)
α/°	90
β/°	90
γ/°	90
Volume/Å^3	2779.33(5)
Z	4
ρ_{calc} g/cm^3	1.309
μ/mm^{-1}	0.828
F (000)	1168.0
Crystal size/mm^3	0.03 × 0.03 × 0.02
Radiation	CuKα (λ = 1.54184)
2Θ range for data collection/°	7.962 to 151.196
Index ranges	$-12 \leq h \leq 12$, $-13 \leq k \leq 14$, $-27 \leq l \leq 27$
Reflections collected	10343
Independent reflections	4888 [R_{int} = 0.0302, R_{sigma} = 0.0403]
Data/restraints/parameters	4888/1/367
Goodness-of-fit on F^2	1.061
Final R indexes [I>=2σ (I)]	R_1 = 0.0361, wR_2 = 0.0945
Final R indexes [all data]	R_1 = 0.0397, wR_2 = 0.0971
Largest diff. peak/hole / e Å^{-3}	0.23/–0.18
Flack parameter	–0.13(10)

Table S2. Crystal data and structure refinement for **4**

Identification code	**4**
Empirical formula	$C_{18}H_{22}N_2O_8$
Formula weight	394.37
Temperature/K	295.75(10)
Crystal system	Tticlinic
Space group	P-1
a/Å	8.09110(10)
b/Å	11.1114(2)
c/Å	11.1400(2)
α/°	83.2370(10)
β/°	75.5170(10)
γ/°	86.0850(10)
Vol µMe/Å^3	942.75(3)
Z	2
ρcalc g/cm3	1.389
µ/mm^{-1}	0.936
F (000)	416.0
Crystal size/mm^3	0.03 × 0.02 × 0.01
Radiation	CuKα (λ = 1.54184)
2Θ range for data collection/°	8.412 to 151.258
Index ranges	–9 ≤ h ≤ 10, –13 ≤ k ≤ 12, –13 ≤ l ≤ 13
Reflections collected	12116
Independent reflections	3710 [R_{int} = 0.0347, R_{sigma} = 0.0297]
Data/restraints/parameters	3710/0/262
Goodness-of-fit on F^2	1.061
Final R indexes [I>=2σ (I)]	R_1 = 0.0496, wR_2 = 0.1582
Final R indexes [all data]	R_1 = 0.0533, wR_2 = 0.1636
Largest diff. peak/hole / e Å^{-3}	0.37/–0.27

Table S3. Important thermodynamic parameters and Boltzmann distributions of the optimized **2** at B3LYP/6-31G(d) level in gas phase

Conformations	Energy (a.u)	Δ G(kcal/mol)	%
**2-1**	-958.352814	0	67.36%
**2-2**	-958.35213	0.43	32.64%

Table S4. The coordinates for the lowest energy conformers of **2**

Energy = -958.35281400 a.u. #Cluster: 1

O	1.11607100	0.52540200	2.69292100
C	1.03578400	-0.28679400	1.52197200
C	2.40700300	-0.43116600	0.85551800
C	2.33066600	-1.21506200	-0.45823100
C	1.27366600	-0.64855800	-1.42026100
C	-2.60225000	-0.35502600	-0.90721400
C	-2.50579200	0.05109400	0.52724100
C	-1.38195600	0.34387500	1.19608800
C	-0.00527400	0.35461700	0.57565500
C	-0.08404600	-0.52854100	-0.71074500
C	-1.25863400	-0.12317600	-1.62009300
C	0.37356900	1.82696100	0.28830800
C	-3.65965100	0.18829100	1.44354200
O	-3.17627100	0.57847000	2.67146500
C	-1.74766300	0.69870300	2.61261800
C	1.22894700	-1.44538800	-2.73121000
C	1.74300000	0.49405900	-2.35282000
O	1.68489700	-0.41079000	-3.50988800
O	-4.83371100	0.01403600	1.22551900
O	0.93451600	-2.55410600	-3.06924500
H	-0.30662100	-1.54948500	-0.36497800
H	1.73387400	0.10211200	3.30869100
H	0.66348100	-1.29021900	1.78827300
H	3.09094700	-0.94158000	1.54682800
H	2.82494200	0.56770100	0.68768500
H	3.31232400	-1.23845500	-0.94528500
H	2.05931300	-2.25845100	-0.25345400
H	-2.88441100	-1.41559200	-0.96417900
H	-3.41316300	0.19314700	-1.40022400
H	-1.17174300	0.93066100	-1.90816100

H	-1.23124200	-0.70727800	-2.54546500
H	1.36045700	1.91803200	-0.17301900
H	-0.35955100	2.29487700	-0.37312500
H	0.39832600	2.39153500	1.22262400
H	-1.30299600	0.02320400	3.34947700
H	-1.46474500	1.72041000	2.88787100
H	1.05535800	1.32731100	-2.49686800
H	2.76259700	0.85517200	-2.19783100

Energy = -958.35213000 a.u. #Cluster: 2

O	1.19209000	0.39269000	2.76077300
C	1.06543500	-0.31067600	1.52668500
C	2.42276100	-0.46735200	0.83630100
C	2.32307100	-1.23907300	-0.48343900
C	1.25343300	-0.66563500	-1.42723800
C	-2.61598400	-0.39256200	-0.84683400
C	-2.49503600	0.01442200	0.58557200
C	-1.36067500	0.31435700	1.23284800
C	0.00374400	0.32880400	0.58878400
C	-0.09356500	-0.55210600	-0.69710200
C	-1.28716000	-0.15544700	-1.58570700
C	0.37152500	1.80198600	0.29423500
C	-3.63183200	0.14255900	1.52477300
O	-3.12684500	0.53249400	2.74365700
C	-1.69994100	0.66055200	2.65861800
C	1.19096800	-1.45075900	-2.74462200
C	1.70886000	0.48565700	-2.35656700
O	1.63515600	-0.40801400	-3.52017400
O	-4.80875200	-0.03705200	1.32823200
O	0.89222400	-2.55601400	-3.08912800
H	-0.30416100	-1.57368300	-0.34553200
H	1.71694000	1.19135800	2.59476900
H	0.69773500	-1.30339400	1.81267000
H	3.10612400	-0.97840500	1.52276600
H	2.85285900	0.52768800	0.65282400
H	3.29755500	-1.26179700	-0.98485400
H	2.05302800	-2.28276200	-0.27967200
H	-2.89396100	-1.45450600	-0.89752400
H	-3.43888700	0.15092800	-1.32492700
H	-1.21174400	0.89806500	-1.87910800
H	-1.27418400	-0.74189100	-2.50988400
H	1.36723200	1.90669300	-0.14671100

H	-0.35063300	2.25868900	-0.38630800
H	0.35173200	2.38771500	1.21869500
H	-1.23664800	-0.01374000	3.38493500
H	-1.42156600	1.68384800	2.93440800
H	1.01870500	1.31988400	-2.48358500
H	2.73038800	0.84644000	-2.21263800
